# Study of novel bidentate heterocyclic amine-based metal complexes and their biological activities: cytotoxicity and antimicrobial activity evaluation

**DOI:** 10.1186/s13065-023-00996-1

**Published:** 2023-07-15

**Authors:** Heba M. Fahmy, Fatma M. Abdel-Rahman, Anwar A. El-Sayed, Ahmed A. El-Sherif

**Affiliations:** 1 Biophysics Department, Faculty of Science, Cairo University, Giza, Egypt; 2 Chemistry Department, Faculty of Science, Cairo University, Giza, Egypt

**Keywords:** AMI complexes, Anticancer activity, Antimicrobial activity, DNA interaction, Antioxidant activity

## Abstract

Metallic antitumor drugs with heterocyclic ligands, such as novel AMI (amino methyl imidazole) complexes [Pd(AMI)Cl_2_](1), [Cu(AMI)L^1^](2), and [Cu(AMI)L^2^·2H_2_O](3) where L^1^ = oxalate and L^2^ = malonate, were synthesized and characterized. Assessments included elemental analyses, mass spectrometry, Fourier transform-infrared spectroscopy, ultraviolet–visible spectroscopy, and thermal analysis. The cytotoxicity of AMI complexes compared to cisplatin was assessed using MTT (3-[4,5-dimethylthiazol-2-yl] 2,5diphenyl tetrazolium bromide) assay with breast (MCF-7) and cervical (HeLa) cancer cell lines. After treating these cells with the AMI complexes' IC_50_ values for 48 h, malondialdehyde levels and catalase activity were used to assess oxidative stress, antioxidant activity was evaluated with DPPH radical scavenging method, comet assays assessed DNA damage, and DNA fragmentation was evaluated using the gel electrophoresis. In vitro, antimicrobial activity was assessed using a disc diffusion method. The anticancer activity results showed that IC_50_ (half-maximal inhibitory concentration) values of complex one, two, and three against MCF-7 and HeLa cancer cells are 0.156 ± 0.0006, 0.125 ± 0.001, 0.277 ± 0.002 μM respectively for MCF-7 cells and 0.222 ± 0.0005, 0.126 ± 0.0009, 0.152 ± 0.001 μM respectively for HeLa cells. Complex two demonstrated strong anticancer activity against MCF-7 and Hela cells. The study of oxidative stress parameters revealed that Malondialdehyde levels increased in cancer cell lines treated with complexes compared to untreated cells. Catalase activity decreased in cells treated with palladium chelate. The DPPH radical scavenging assay results identified that complex one was a more potent antioxidant in MCF-7 and Hela cells than other complexes with SC_50_ values of 227.5 ± 0.28 and 361 ± 1.2 μL/mL, respectively. The comet assay results showed that complex two caused significant DNA damage in MCF-7 and HeLa cancer cells treated. Antimicrobial assays identified complex three as the most effective. Copper complexes give better antifungal activity against *A. flavus* than the palladium complex. We conclude that complex two is the most active in both cell types and might be assessed as a clinically useful drug for breast cancer treatment. The significance of the current study is the synthesis of antitumor drugs containing heterocyclic ligands, such as novel AMI complexes, and the study of their biological activities.

**Supplementary Information:**

The online version contains supplementary material available at 10.1186/s13065-023-00996-1.

## Introduction

Metal ions play important roles in biological processes. The field of knowledge concerned with the application of inorganic chemistry to the therapy or diagnosis of a disease is medicinal inorganic chemistry. Introducing metal ions or metal ion binding components into a biological system for treating diseases is one of the main subdivisions in bioinorganic chemistry. Intentionally introducing metal ions into the human biological system has proven useful for diagnostic and therapeutic purposes [1].

One of the most severe and complicated threats to human life is cancer, caused by the rapid formation and proliferation of abnormal cells that can spread beyond their natural boundaries [2]. According to the WHO (World Health Organization), 18.1 million individuals worldwide were diagnosed with cancer in 2018, and 9.6 million died from it [3]. As a result, researchers have been searching for alternative treatments and more effective and selective drugs to treat cancer [4]. Anticancer drug development is one of the biological studies' most quickly evolving fields [5]. Increasing the range of metal-based drugs is critical for increasing activity and inducing apoptosis in various tumor cells [6–8]. Among these drugs, Platinum (Pt) anticancer drugs are commonly used in clinical therapy, particularly in testicular, ovarian, and neck and head cancers [9, 10]. Their ability to bind to DNA covalently and modify its structure gives platinum agents their anticancer effects, influencing cellular processes and ultimately leading to cancer cell death [11, 12]. Cisplatin [Cis diamminedichloroplatinum(II)] was the first platinum anticancer drug that entered clinical trials [13–15]. Cisplatin and its analogs' anticancer activity are mainly attributed to a specific intrastrand adduct formed by platinum crosslinking N7 atoms of nearby guanine residues in DNA. It was found that the cisplatin analogs' anticancer effects are modulated by the carrier amine ligands [16]. Transplatin, unlike cisplatin, cannot form intrastrand in double-helix DNA between consecutive base residues due to steric reasons.

Nevertheless, if two bases on the same strand are separated by at least one intervening base, transplatin can crosslink them [17]. The synthesis of platinum(II) complexes with numerous ligands has been the target of much research. However, other metal complexes soon followed; some metals were used as an effective chemotherapeutic, antibacterial, and antifungal drug [18]. In the design of metal-based chemotherapeutic agents, the choice of metal ion and the effect of the ligand is crucial [19]. A heteroatom like nitrogen, sulfur, or oxygen is commonly used to bind metals with ligands. A coordination complex is created when a coordination bond is formed between the metal and the ligand; metals and their complexes have played essential roles in medicine for over 5000 years [20, 21].

Metal complexes' unique structure, reactivity, and ease of synthesis have made them effective ligands in drug development research [22]. As a drug crosses the cell membrane and enters the cell, the structure and arrangement of the ligands may influence the drug's mode of action and metabolism [23]. Metal complexes' pharmacological activity is determined by the coordination of metal ions and ligands [24–26]. Rohand et al. [27] reported that the antimicrobial activity in vitro reveals that metal complexation increases the complexed ligand's antibacterial activity *versus* both bacterial strains compared to the free ligand (Complexes' higher activity could be attributed to their increased lipophilicity due to their chelation [28]. Although cisplatin and its derivatives have been widely utilized to treat certain malignancies, their therapeutic efficacy is limited by systemic toxicity and resistance [29–32]. For these limitations, scientists are searching for and synthesizing novel metal complexes with enhanced anticancer activity while reducing toxicity and resistant properties [33–39]. Metal complexes comprising a variety of imidazole-containing ligands were widely explored as model compounds of metal active sites. Among these complexes, copper(II) complexes containing heterocyclic amines have significant importance because they demonstrate a variety of biological activities, including anticancer activity [40] and antimicrobial activity [41]. Palladium(II) complexes of imidazoles have been shown to have potent antimicrobial and antitumor activities [42–44]. The binding of imidazole to transition metal ions is of great interest, particularly in biological systems [45, 46]. Imidazole derivatives consider the basic fragment of various natural products and biological systems. The imidazole ring acts as a co-ligand in metal complexes, possibly allowing them to bind to biomolecules [47]. Because of the significant biological activity of imidazole and its derivatives, particularly as antiprotozoal, antifungal, and antihypertensive agents, they have a unique place in medicinal chemistry [48, 49].

The complexes, [Imz-H][Fe(pda)2]0.1.3H_2_O (1) and [Mn(Imz)6]. 2Cl^−^0.2H_2_O (2), where Imz = imidazole and H_2_pda = 2,6 pyridine dicarboxylic acid, were synthesized by Khan et al. [50]. The antimicrobial activity of complexes was studied in vitro using the disc diffusion method versus some bacteria such as *Staphylococcus aureus, Escherichia coli, Klebsiella pneumonia,* and *Listeria monocytogenes* and some fungi like *Fusarium moniliform* and *Candida albicans*. It was revealed that complexes had the highest antibacterial activity against *E. coli*, *S. aureus,* and *K. pneumonia* but showed low activity versus *L. monocytogenes*. Complexes showed more potent activity against Candida albicans than Fusarium moniliform for the antifungal activity. The cytotoxicity of complexes was studied in vitro by the MTT assay against a human macrophage/monocyte cell line. Results revealed that complex 1 at IC50 = 7.89 mM had higher cytotoxic activity than complex 2 at IC50 = 15.98 mM. Abdel-Rahman et al. [51] synthesized the following copper complexes Cu(Bzphe)_2_(Imi)_2_(OH_2_)_2_ (1) Cu(Bzphe)_2_(Mimi)_2_(OH_2_)_2_](2), Cu(Bzphe)_2_(Bipy)(OH_2_)_2_(3), and Cu(Bzphe)_2_(Phen)(OH_2_)_2_ (4) where Bzphe = *N*-benzoyl-dl-phenylalanine, Imi = imidazole, Mimi = methyl imidazole, Bipy = 2,2′-bipyridine and Phen = 1,10-phenanthroline. The biological activity, such as antimicrobial activity, was studied in vitro versus two Gram-positive bacteria (*Bacillus subtilis* and *Micrococcus luteus*), one Gram-negative bacteria (*Escherichia coli*), and three fungal cultures (*Aspergillus niger, Candida glabrata*, and *Saccharomyces cerevisiae*). Results showed that copper complexes significantly inhibit pathogens' growth in this study. Also, results showed that complex 3 had the highest activity and activity of complexes affected according to this order CuImiBzphe < CuMimiBzphe < CuPhenBzphe < CuBipyBzphe. In the end, it was found that the synthesized complexes showed good antimicrobial activity. Fnfoon and Al-Adilee [52] studied the complexation of Cu(II), Ag(I), and Au(III) ions with newly synthesized imidazole ligand, which was prepared by a coupling reaction between 2-]2′-(4-Chloro Phenyl)azo[-1-Methyl imidazole]. The anticancer activity of these complexes is assessed by MTT assay against brain cancer (A172) cell lines. Results showed that synthesized complexes demonstrated effective anticancer activity and provided great potential as anti-tumor medication options in the future. This study aims to synthesize antitumor drugs containing heterocyclic ligands such as novel AMI complexes through a chelation process to avoid converting *trans* form, which is clinically ineffective as cis and *transplatin* [53–55]. Also, the cytotoxic effect of these metal complexes on the breast (*MCF-7*) and cervical (*HeLa*) cancer cell lines was studied. Additionally, the study's aim extends to the study of the complexes-DNA interaction. Also, the prepared complexes' antimicrobial and antioxidant activities were studied in vitro as in our previously published work [56–58] (Additional file 1).

## Materials and methods

### Materials

#### Chemicals and reagents

All chemicals utilized were of the greatest analytical purity. They included 2-(amino methyl imidazole) = (AMI), oxalic acid, and malonic acid that Sigma-Aldrich supplied. The metal salts of CuCl_2_·2H_2_O (Sigma-Aldrich) and K_2_PdCl_4_(Merck) were used. Organic solvents such as ethyl alcohol (99%) and dimethylformamide (DMF), in addition to dimethyl sulfoxide (DMSO), were used. All preparations are usually made use of de-ionized water collected from glass equipment.

#### Solutions

Fresh stock solutions of 1 × 10^−3^ M binary and mixed ligand complexes were prepared by dissolving the chelates in a sufficient volume of ethanol with exact weight. After that, all the complex solutions' conductivity was measured. The recommended procedures standardized the metal salt solutions [59, 60]. The previous stock solutions were diluted to prepare different complexes' solutions that could be used to study UV–Vis spectra.

#### Cell lines

MCF7 cells: the human epithelial breast carcinoma cell lines, and HeLa cells: the human cervical cancer cell line, was imported from ATCC (The American Type Culture Collection; Manassas, VA, USA).

### Methods

#### Synthesis of metal complexes

**Fig. 1:**
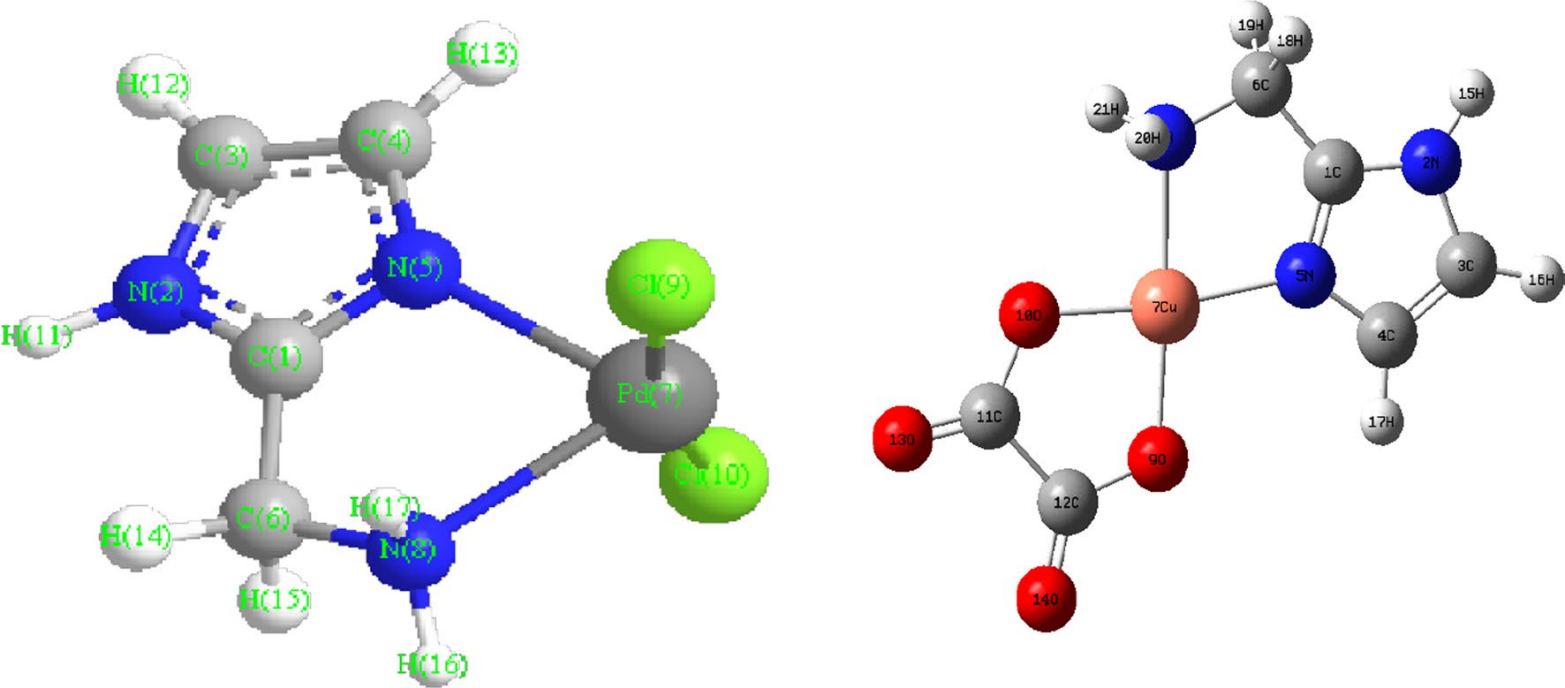
The structures of [Pd(AMI)Cl_2_] and [Cu(AMI)L^1^], where AMI = amino methyl imidazole, L^1^ = oxalate

The complex binary was prepared by mixing a hot ethanolic solution of AMI ligand (1 mmol, 0.170 g) and 0.326 g K_2_PdCl_4_. A hot solution of ethanol (20 mL) (70 °C) of AMI (1 mmol, 0.170 g) as the primary ligand was mixed with a hot solution of ethanol of oxalic acid (1 mmol, 0.104 g) or malonic acid (1 mmol, 0.104 g) as a secondary ligand with metal chloride salt (1 mmol, 0.170 g CuCl_2_·2H_2_O) dissolved in absolute hot ethanol (20 mL) (70 °C) for preparation of the mixed ligand chelates (complex two and complex three, respectively). After two hours of reflux stirring of the resultant mixture, the complexes precipitated. Filtration was used to collect the sample, washed numerous times in diethyl ether for purification before being dried under a vacuum over anhydrous calcium chloride. Pure mixed ligand complexes were obtained by recrystallization from ethanol (Fig. 1).

#### DFT calculation

**Fig. 2:**
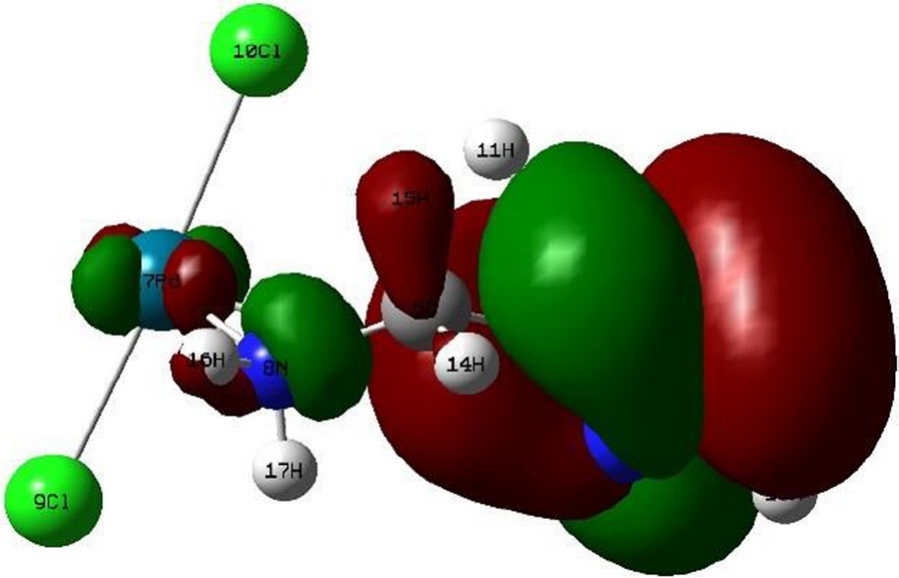
HOMO of [Pd(AMI)Cl_2_], where AMI = amino methyl imidazole

**Fig. 3:**
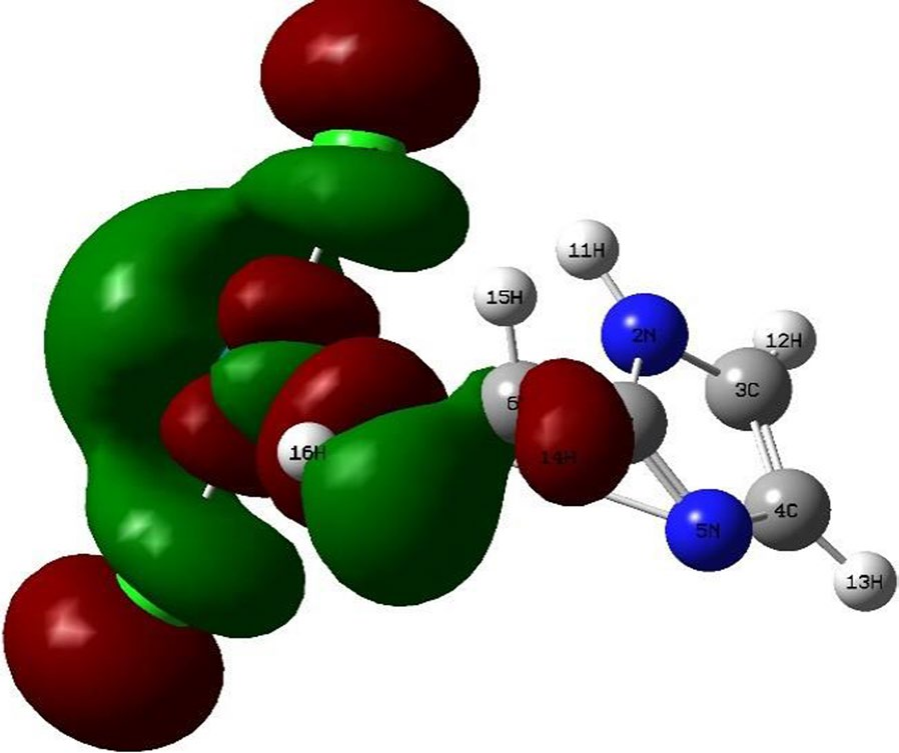
LUMO of [Pd(AMI)Cl_2_] where AMI = amino methyl imidazole

**Fig. 4:**
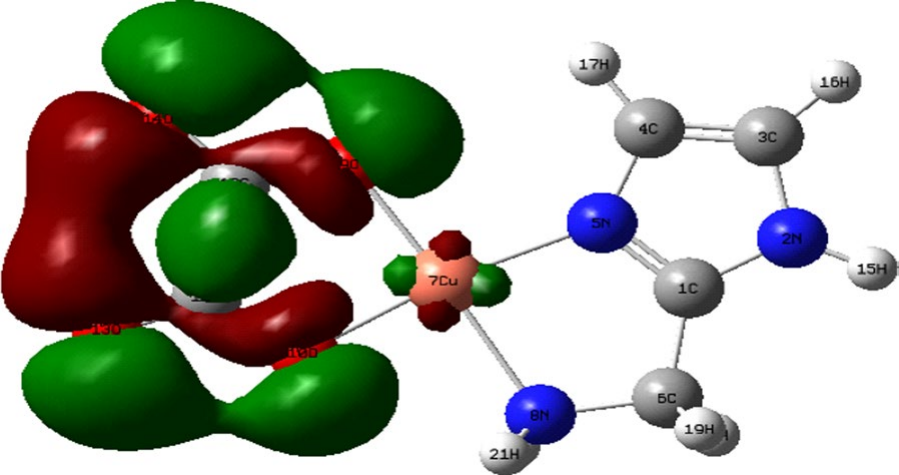
HOMO of [Cu(AMI)L^1^], where AMI = amino methyl imidazole, L^1^ = oxalate

**Fig. 5:**
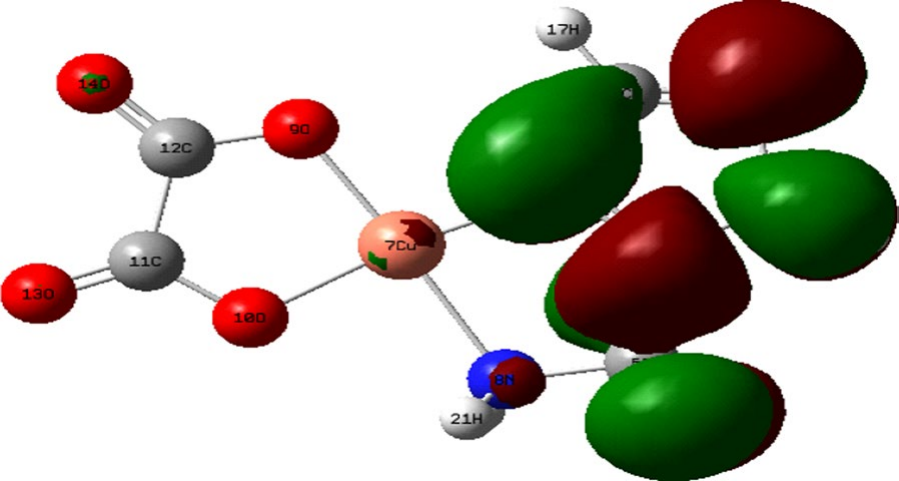
LUMO of [Cu(AMI)L^1^], where AMI = amino methyl imidazole, L^1^ = oxalate

Density Functional Theory (DFT) calculations by the program Gaussian 09 at the B3LYP/LANL2DZ level of theory were used to optimize the structure of the investigated complexes. Different configurations and compositions of coordination's spheres are used for complexes, and the configurations with the lowest total energy were chosen [61] (Figs. 2, 3, 4, 5).

#### Characterization of newly prepared AMI complexes

Newly prepared AMI complexes were subjected to elemental analyses, EI-mass, IR, UV–Vis, and thermal analyses. Elemental analyses and molar conductivity measurements in which the new binary and ternary complexes of AMI were prepared in 1:1 and 1:1:1 molar ratio for complex one and complex (two, three), respectively, and at 25 ± 2 °C, the molar conductivity values (Λ_m_) of the different complexes in DMF solvent (10^–^^3^ M) were found [59, 62]. The molar conductivity was measured using a Jenway 4010 conductivity meter. Carbon, hydrogen, and nitrogen microanalyses were performed with a CHNS-932 (LECO) Vario elemental. A triforce XMTD-3000 was used to evaluate the melting point. The electron ionization technique at 70 eV with an MS-5988 GS-MS Hewlett-Packard apparatus was used to record the mass spectra of the prepared complexes [63]. The various chelates' IR spectra in the KBr disc in the 4000–400 cm^−1^ range were evaluated on a PerkinElmer 1650 spectrometer to assess the different ligands-metal ions' binding modes in the chelates and compared to the free ligands [64]. The synthesized chelates' UV–vis spectra in DMF solvent were measured by automated spectrophotometer UV–vis Perkin–Elmer Model Lambda ranging from 200 to 700 nm at room temperature [65]. The thermogravimetric (TG) and differential thermogravimetric (DTG) tests of solid complexes were made with a heating rate of 10 °C min^−1^ from 30 to 1000 °C on a Shimadzu TG-50H thermal analyzer [66].

#### Cytotoxicity (MTT assay)

The MTT assay is a colorimetric method for evaluating cell viability that is sensitive, quantitative, and repeatable. The assay is focused on the ability of mitochondrial lactate dehydrogenase enzymes (LDH) in living cells to transform the water-soluble substrate 3-(4,5-dimethylthiazol-2-yl) 2,5 diphenyl tetrazolium bromide (MTT) into a dark blue water-insoluble formazan. To dissolve the insoluble purple formazan product into a colored solution, a solubilization solution (dimethyl sulfoxide) is added. The absorbance of this colored solution can be measured with a spectrophotometer at a wavelength of 570 nm [67]. The cytotoxic effect of metal complexes and cisplatin as positive control on cancer cell lines, specifically the human breast cancer cell line (MCF-7) and cervical cancer cell line (HeLa), was assessed by the MTT assay. These cells were cultured in Ninety-six well plates (10,000 cells/well) and lifted for 48 h for attaching. Both types of precultured cell lines were exposed to different concentrations of metal complexes and a controlled drug (cisplatin) for 48 h at 37 °C using a two-fold serial dilution procedure. Treated cell lines were examined microscopically to detect morphological changes and detached cells. Phosphate-buffered saline (pH 7.2 0.2) removed dead cells (PBS-0.05 percent Tween). Residual live cells were stained with 0.5% MTT at a 25 μL/well concentration. For 3–4 h, plates were incubated at 37 °C. For 30 min on a plate shaker, 0.05 mL DMSO was used to dissolve newly formed intra-cytoplasmic MTT formazan crystals. The IC_50_ values of metal complexes and control were calculated using the Master-plex-2010 program [68, 69].

Using the MTT assay, the viability of the cells was evaluated where optical densities were evaluated at 570 nm using an ELISA plate reader (Biotek—8000, USA). The viability percentage was calculated as follows: Cell viability percentage = (OD of treated cells/OD of untreated cells) × 100 [70, 71].

#### Antimicrobial activity

The synthesized complexes' biological activity was assessed by a modified Kirby–Bauer disc diffusion method in vitro [72] using six bacteria: three Gram-negative: *Escherichia coli, Neisseria gonorrhoeae,* and *Pseudomonas aeruginosa* and three Gram-positive: *Bacillus subtilis, Staphylococcus aureus,* and *Streptococcus faecalis* and using two fungi: *Aspergillus flavus* and *Candida albicans*. Standard Ampicillin (Antibacterial agent) and Amphotericin B (Antifungal agent) were positive controls for antimicrobial activity, while filter discs impregnated with 10 µL of solvent (DMSO) were used as a negative control. Using the disc diffusion process, every pathogenic organism's isolated colony should be chosen from primary agar plates and checked for susceptibility [73]. The species are cultured on a nutrient agar medium placed in a petri dish. After that, 10 µ of the stock solution concentrations tested were loaded on sterile paper discs with a diameter of 8.0 mm. When a chemical is impregnated, a filter paper disc is mounted on agar, and the chemical diffuses from the disc into the agar [74]. Due to this diffusion, the chemical would only be present in the agar around the disc. The chemical's solubility and molecular size were used to determine the chemical infiltration area around the disc. An organism placed on the agar will not grow in the area around the disc if it is susceptible to the chemical. This area of no growth around the disc is called the inhibition zone. The diameters of this zone were measured after gram-positive and gram-negative bacteria were incubated at 35–37 °C for 24–48 h and fungi were incubated at 30 °C for 24–48 h [72] using slipping calipers from the National Committee for Clinical Laboratory Standards [73]. Disc diffusion methods are simpler and faster than broth-based methods [75, 76].

#### Determination of oxidative stress markers

A commercially available kit measured catalase activity and Malondialdehyde (MDA) levels.

##### Determination of catalase activity

One of the body's defense mechanisms versus hydrogen peroxide (H_2_O_2_) is catalase, an antioxidant enzyme in most aerobic cells, where H_2_O_2_ is a powerful oxidant that causes intracellular damage. At 25 °C and pH 7.0, each unit of catalase decomposes 1 μM of H_2_O_2_ per minute. Catalase assay is considered an antioxidant Biomarker; in addition to that, it provides another valuable tool for oxidative stress research [77]. The catalase activity was measured spectrophotometrically using the Aebi method [78], in which catalase interacts with a specific amount of H_2_O_2_. With a catalase inhibitor, the reaction is halted in exactly one minute. When peroxidase (HRP) is present, the residual H_2_O_2_ interacts with 3,5-Dichloro-2-hydroxybenzene sulfonic acid (DHBS) and 4-aminophenazone (AAP) to generate a chromophore with a color intensity that is inversely proportional to the amount of catalase in the original sample. Then, the chromophore color's absorbance was recorded at 510 nm.

##### Determination of lipid peroxidation

Malondialdehyde (MDA) levels were used to assess lipid peroxidation, and the assessment of lipid peroxidation in cells is a vital sign for determining the extent of oxidative stress [79]. Ruiz-Larrea et al.'s [80] method assessed MDA by measuring thiobarbituric reactive species. In this method, the tubes that contained the samples and standards were prepared, and TBA solution was added to each tube and mixed well. Then heat it in a boiling water bath for 30 min and then cool in ice bath for 10 min. In this assay, the Malondialdehyde in the samples interacts with thiobarbituric acid (TBA) in an acidic medium at 95 °C to generate the thiobarbituric acid reactive product. A spectrophotometer measures the pink product's absorbance at 534 nm. The intensity of the color indicates the MDA concentration [81].

#### Measurements of antioxidant activity utilizing the DPPH radical scavenging method

Free radical scavenging activity of newly prepared AMI complexes in MCF-7 and HeLa cancer cell lines was assessed by 1, 1-diphenyl-2-picryl hydrazyl (DPPH). Due to its odd electron paramagnetic properties, DPPH is a stable radical in this assay [82]. In a nutshell, a 0.1 mM DPPH solution was prepared in ethanol, and 1 m from this solution was then added to 3 mL of different supernatant from cancer cell lines (treated and controlled) in ethanol at different concentrations (3.9, 7.8, 15.62, 31.25, 62.5, 125, 250, 500, 1000 μL/mL). The mixture was vigorously mixed and set aside for 30 min at room temperature. During this period, the DPPH radical became diamagnetic when it could accept an electron from sample molecules [82]. Then, a spectrophotometer (UV–Vis Milton Roy) measured the absorbance at 517 nm. The Log dose inhibition curve was used to compute the samples’ SC_50_ value, defined as the samples' concentration that can inhibit the DPPH free radical by 50% [83]. Using the following equation, the % DPPH scavenging effect was calculated: DPPH scavenging effect (%) or Percent inhibition = $\frac{{A}_{C}-{A}_{S}}{{A}_{C}}$  × 100%. Where A_C_ is the absorbance of DPPH only and A_S_ is denoted by the absorbance in the presence of tested samples.

#### DNA comet assay (single cell gel electrophoresis)

The comet assay, the alkaline single-cell gel electrophoresis assay [84], identifies DNA strand breakage at the single-cell level with significant sensitivity. The comet test is a commonly used technique to determine whether or not chemical substances are genotoxic [85–88]. The Comet assay measured the DNA damage in MCF-7 and HeLa cell lines after treating these cells with newly prepared AMI complexes for 48 h. The comet assay s alkaline version was performed following the method reported by Singh and his collaborators with minor alterations [84]. At 35–45 °C, 10,000 treated and untreated cells were suspended in a final concentration of 0.5–1.0% LMPA (Low Melting Point Agarose) and allowed for at least two minutes before placing all those cells on a microscope slide. The slides were allowed to harden for a short time (5 min) at 4 °C and covering with a coverslip, After that, the slides were immersed in an ice-cold alkaline lysis buffer (2.5 M NaCl, 10 mM Tris, 100 mM EDTA, 1% Triton X-100, final pH 10.0) for 1 h at 4 °C so, the cells were lysed, because the lysing solution would break down the cell membrane, and also it would release the DNA from histone for studying DNA damage by this method, after that removing the slides from lysis solution and then immersed it in Electrophoresis Buffer (alkaline buffer(0.3 M NaOH, 1 mM EDTA, pH > 13) for 20 min to enable DNA unwinding and the expression of alkali-labile damage and electrophoresis (0.74 V/cm, 300 mA, 30 min) was then performed and then immersing the slides in 70% ethanol for 5 min before being removed, tapped, and left to dry at room temperature and following that, the slides were stained with 80L 1X Ethidium Bromide, left for 5 min and to remove excess of dye then, placing it in distilled water, All of the preceding procedures were carried out in dark illumination to avoid any additional DNA damage caused by sunlight. To evaluate DNA damage, observations of EtBr-stained DNA were performed using a 40 × objective on a fluorescent microscope and subsequently utilizing Kinetic Imaging, Ltd.'s Komet 5 image processing software(Liverpool, UK) for studying DNA in the cell by calculating the percentage of migrated DNA, the length of DNA migration, %DNA in tail and also measured tail moment. In most cases, 50 to 100 cells were randomly selected and evaluated per slide [84, 89–95].

#### DNA extraction from cancer cell lines

A 1.5 mL microfuge tube added 0.5% SDS extraction buffer to 200 μL (around 1 × 10^4^–5 × 10^6^ cells) of cell suspension (treated and untreated). Then, 50μL of proteinase-K solution (10 mg/mL) was added. After that, the tubes were mixed and incubated for 12 h at 55 °C for 3 days with occasional vigorous mixing. Following that, the tubes were removed from the incubator, and each tube was applied to a vortex for 2–5 s after adding 0.7–0.8 mL (phenol: chloroform: isoamyl alcohol in the ratio of 25:24:1). Then, the samples' centrifugation was performed for 3–5 min at 12.000 rpm. Subsequently, from each sample, 400–500 μL of the aqueous layer was removed and placed in a new tube, and each tube was then filled with 40–50 μL 3 M sodium acetate pH 5.3 100% ethanol added until the 1.5 mL mark was reached. For DNA precipitation, each sample tube was set aside at − 20 °C overnight and subsequently centrifuged again for 20 min at 12.000 rpm, and then the supernatant and any excess fluid were removed. The DNA pellet was extracted from treated and untreated cells. It was allowed to be dissolved in 50 μL of tris EDTA buffer and then processed to agarose gel electrophoresis for migration distribution analysis [96].

#### Agarose gel electrophoresis of extracted DNA

Identical amounts of DNA (10 μL) were extracted from control and treated cell suspension samples and were combined with two μL of gel-loading dye. The prior mixture was then poured into the wells of the solidified gel. The DNA electrophoresis was performed at 50 V for around 2 h on a 1% agarose gel in Tris–acetate EDTA (TAE) buffer containing 0.1 mg/mL ethidium bromide (EtBr) for staining samples. The image analysis software was used to analyze DNA sample migration distribution [96].

#### Statistical analysis

The data regarding antimicrobial activity, oxidative stress parameters, and comet assay parameters were presented as means ± S.E.M. To compare statistical differences (*P*-value) between treated and untreated samples data of comet assay and oxidative stress parameters, using SPSS to run a one-way analysis of variance (ANOVA) with the least significant difference (LSD) test. The statistically significant difference is denoted by *p* < 0.05. The given data's percentage difference (D %) was also calculated as follows:

$$ D\% = \frac{treated\,value - control\,value}{{control\,value}} \times 100\% . $$

## Results and discussion

### Characterization of AMI complexes

#### Elemental analyses and molar conductivity measurements

New binary and ternary AMI complexes were prepared in 1:1 and 1:1:1 molar ratios for complexes one, two, and three. Elemental analysis results (C, H, N) agree with the formulas in Table 1. Complexes display sharp melting points indicating their high purity. Elements (C, H, N, and metal content), melting points, molecular formulas, and compound yields are listed in Table 1 of the compound study. Different Complexes in DMF solvent (10^−3^ M) exhibit molar conductance of 9–26 Ω^−1^ mol^−1^ cm^2^ at 25 °C ± 2 °C. These low values indicate a non-electrolytic nature. Thus, chloride is located in the coordination sphere in a complex one, and the secondary ligand likely behaved as a dinegative, bidentate moiety [62, 97, 98]. The results are tabulated in Table 1.

**Table 1:** Physical and analytical data of AMI binary and ternary metal complexes

Complex no.	Compound (chemical formula)	Color yield (%)	M.p. (°C)	Found (Calcd)				A_m_ (Ω^−1^ mol^−1^ cm^2^)
				C (%)	H (%)	N (%)	M (%)	
1	[Pd(AMI)Cl_2_]	Pale yellow (92)	> 300	14.38 (17.51)	2.17 (2.55)	15.05 (15.30)	38.59 (38.80)	26
2	[Cu(AMI)L^1^]	Brown (89)	260	28.88 (28.97)	2.78 (2.82)	16.70 (16.90)	25.12 (25.35)	9
3	[Cu(AMI)L^2^·2H_2_O]	Brown (88)	190	27.99 (28.14)	4.11 (4.36)	13.77 (14.07)	21.03 (21.10)	11

AMI = amino methyl imidazole, L1 = oxalate, L2 = malonate, A_m_ = molar conductance, and M.P. = melting point

#### Mass spectral studies

**Fig. 6:**
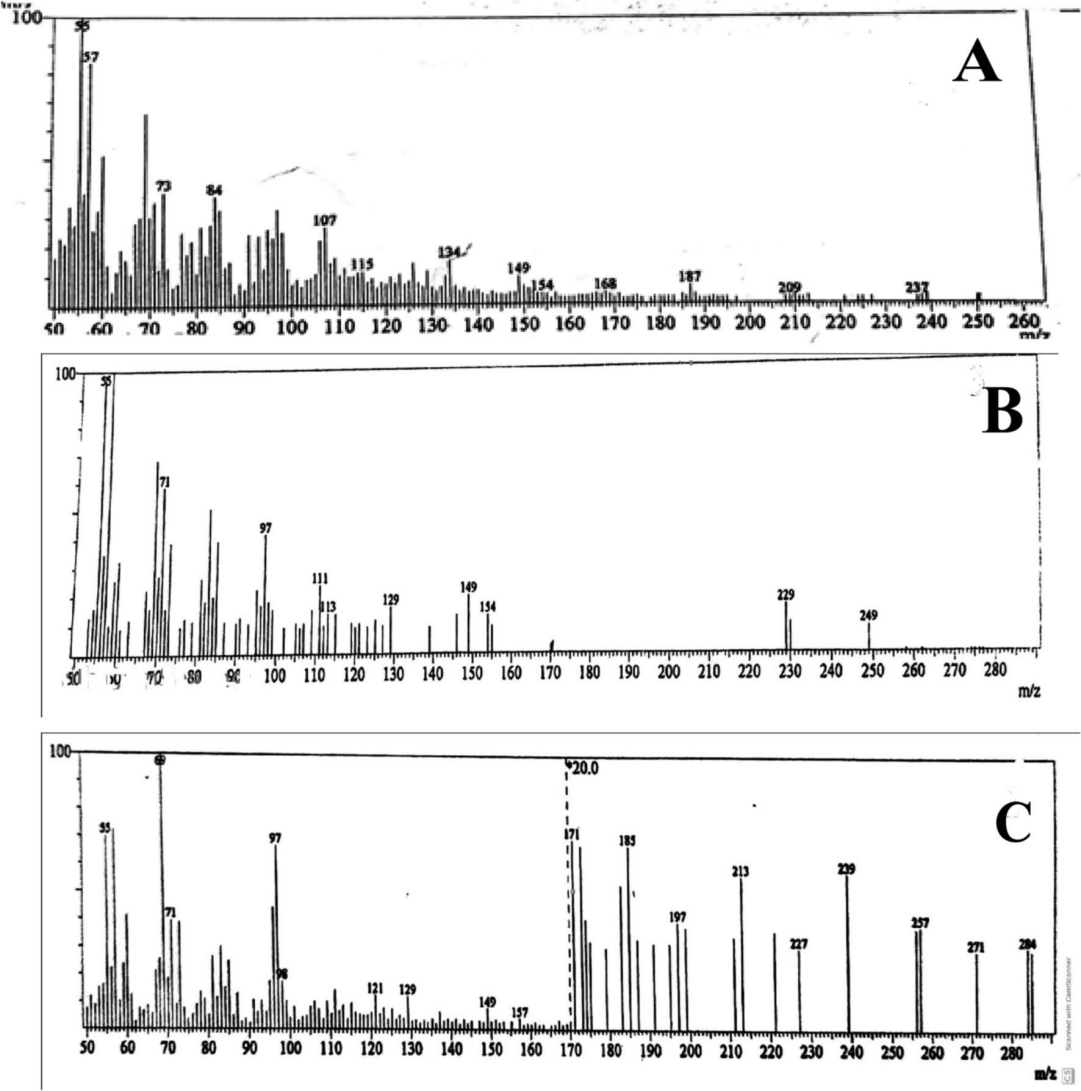
Mass spectra of different complexes. **A** Complex one [Pd(AMI)Cl_2_], **B** complex two [Cu(AMI)L^1^] and **C** complex three [Cu(AMI)L.^2^·2H_2_O]

The mass spectra of complexes 1, 2, and 3 as representative complexes (Fig. 6), Complexes 1, 2 and 3 provide good evidence for the molecular formulas from their highest mass peak at m/z 250, 249, and 280, respectively, which agree well with the proposed formula weights for the non-hydrated or non-solvated complexes. All complexes' mass spectra reflected the presence of the AMI (primary ligand) at m/z 170 (calcd. 170.4 g/mol) [63, 99, 100].

#### IR spectral analysis

**Fig. 7:**
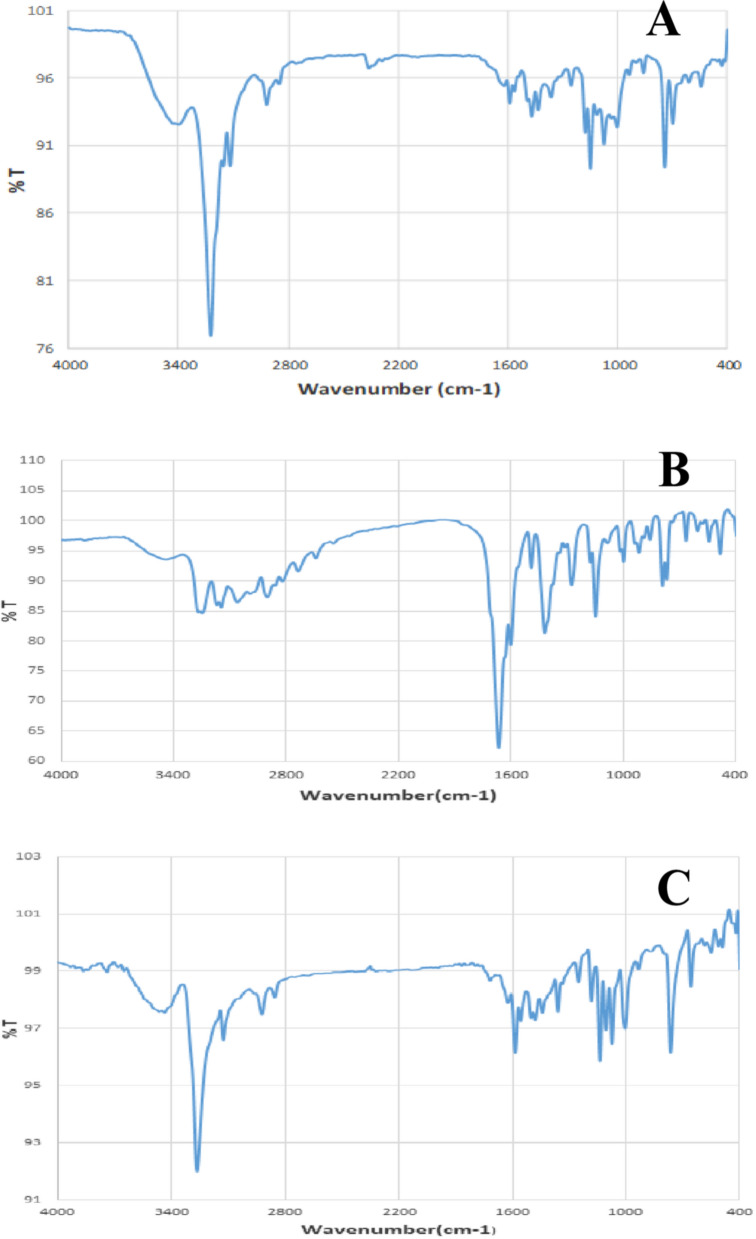
IR spectra of complexes. **A** Complex one (Pd(AMI)Cl_2_), **B** complex two (Cu(AMI)L^1^) and **C** complex three (Cu(AMI)L^2^·2H_2_O). Where T: transmittance

IR measurements show a sharp band at 3230 cm^−1^ attributed to free AMI (NH_2_) stretching vibration (Table 2 and Fig. 7). This band shifted in all complexes. Small intensity bands at 611–676 cm^−1^ were attributed to ρ(NH_2_) bending vibrations, further supporting the coordination of NH_2_ nitrogen atoms with metal ions [64]. IR spectra of complexes two and three showed an absence of OH group bands, confirming the deprotonation of malonic and oxalic acids during coordination [101]. IR spectrum of the parent AMI ligand showed stretching vibrations of *υ*(C=N) of the imidazole ring at 1562 cm^−1^. The ν(C=N) stretching vibration shifted to higher frequencies, 1585–1602 cm^−1^, in the complexes, which may imply that the *υ*(C=N)_imidazole_ nitrogen participates in coordination (Table 2).

**Table 2:** Important infrared bands of AMI binary and ternary metal complexes

No.	Compound	*υ*(NH_2_) (sh)	*υ*(C = N)_*imidazole ring*_	(*COO*)*Asy* (s)	(*COO*)*sym*	*υ*(*CO*) (m)	(*NH*_2_)_*bending*_	*υ*(M–O)	*υ*(M–N)
1	[Pd(AMI)Cl_2_]	3225	1590 m	–	–	–	611w	–	455w
2	[Cu(AMI)L^1^]	3262	1585 s	1492	1342 s	1277	657w	547w	485w
3	[Cu(AMI)L^2^·2H_2_O]	3267	1602 m	1501	1359 m	1249	676 s	550 s	419 s

sh = sharp, m = medium, s = small and w = weak

The AMI ligand behaved as a neutral bidentate ligand coordinated with metal ions via imidazole and NH_2_ nitrogens. In contrast, malonic and oxalic acids functioned as uni-negative, bidentate ligands via carboxylate O and amino N. The spectra of complexes two and three showed bands at 1492–1502 and 1342–1359 cm^−1^, corresponding to ν(COO)_asy_ and ν (COO)_sym_ groups, respectively. This band shift may imply that the secondary ligand is coordinated with metal ions via carboxylate oxygen [102, 103]. New bands at 419–485 cm^−1^ and 547–550 cm^−1^ appeared when M–N and M–O form in spectra of various chelates.

#### The UV–vis spectral studies

**Fig. 8:**
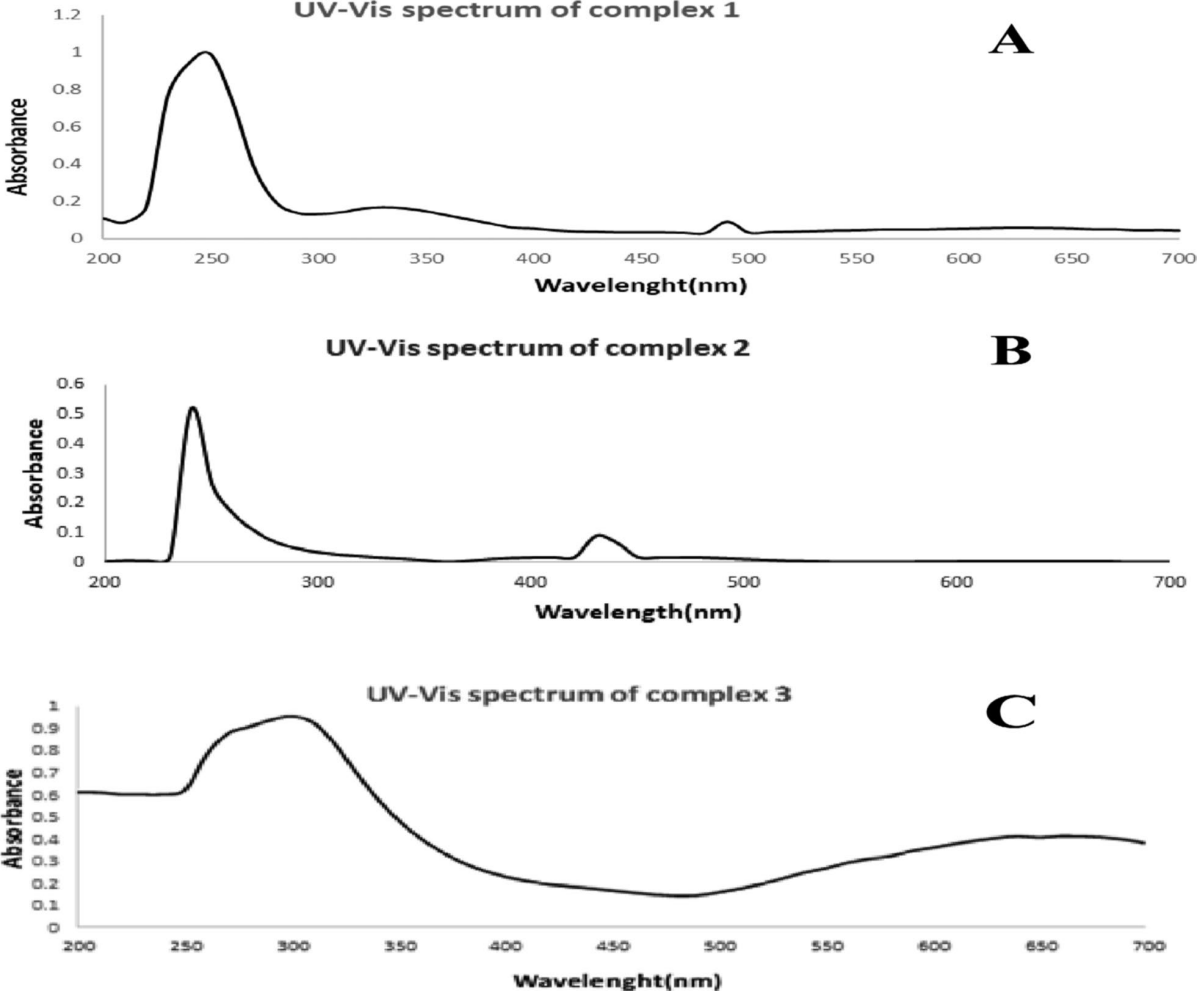
Metal complexes' UV–Vis spectra. **A** Complex one (Pd(AMI)Cl_2_), **B** complex two (Cu(AMI)L^1^) and **C** complex three (Cu(AMI)L^2^·2H_2_O)

The electronic spectra in DMF solution (Table 3 and Fig. 8). showing absorption of high energy 243–250 nm and 320–342 nm are assigned to the intraligand π → π and n → π. π → π transitions respectively. For complex 1 and complex 2, the intraligand charge transfer band appeared at 490 and 418, respectively [65, 104, 105]. ‏

**Table 3:** UV–Vis transitions of AMI binary and ternary metal complexes

Transition	[Pd(AMI)Cl_2_](1)	[Cu(AMI)L^1^](2)	[Cu(AMI)L^2^·2H_2_O](3)
π–π*	250	243	250
n–π*	342	Disappear	320
Charge transfer	490	418	–

#### Thermal studies

**Fig. 9:**
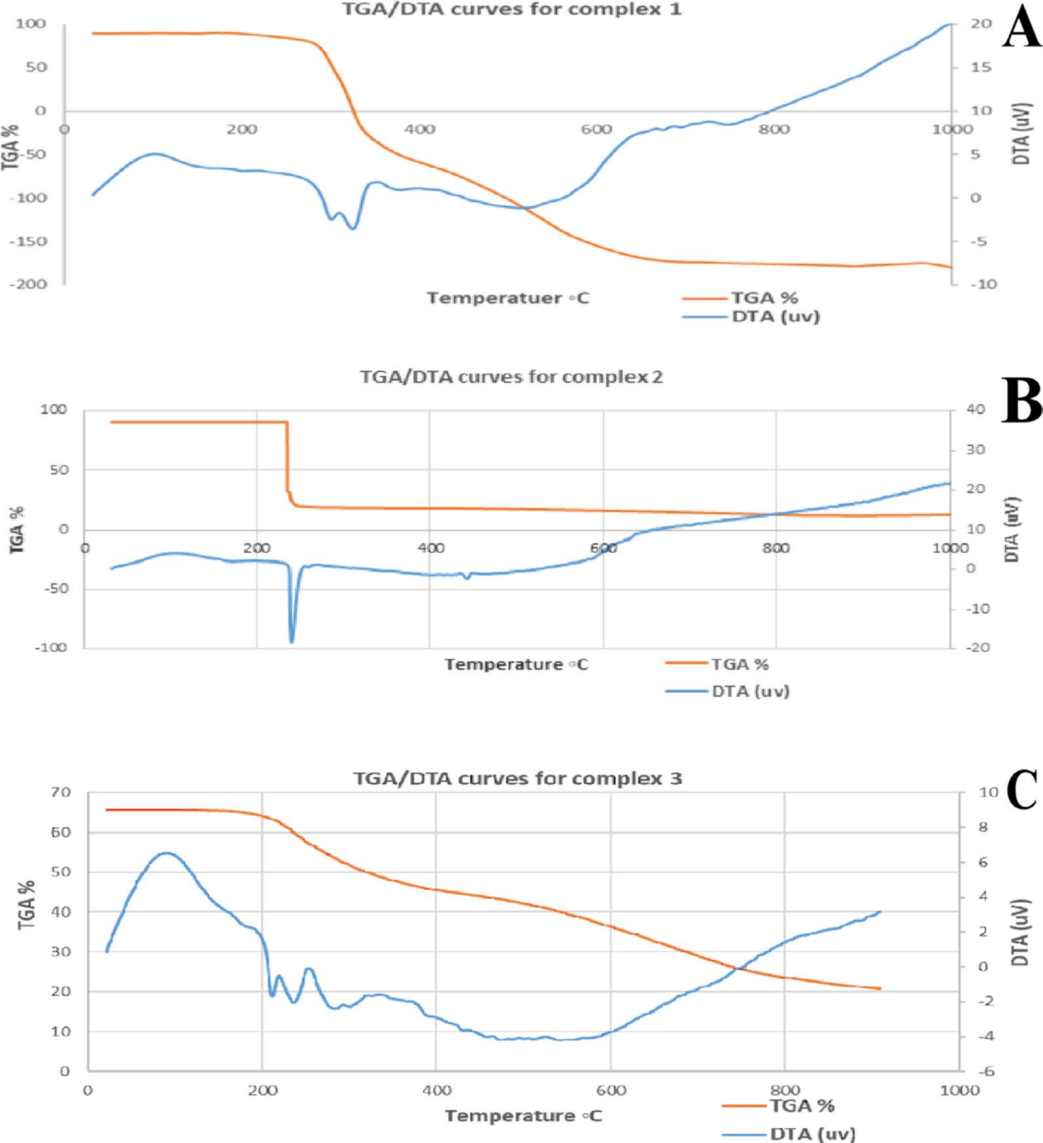
TGA/DTA curves for newly prepared AMI complexes. **A** Complex one [Pd(AMI)Cl_2_], **B** complex two [Cu(AMI)L^1^] and **C** complex three [Cu(AMI)L^2^·2H_2_O]

TG and DTG techniques are useful for determining the thermal stability of Schiff base ligands and mixed ligand chelates to assess the thermal decomposition behavior of new complexes. The TG and DTG results were tabulated in Table 4. Complex one (Pd(II) chelate) decomposed in three stages (Fig. 9). The first two decomposition stages occurred at 145–465 °C. These stages might be 2HCl gas and HCN molecule loss with a mass loss of 36.95% (calcd 36.43%). The third stage, at 465–730 °C, reflects C_2_H_4_N_2_ molecule loss with a mass loss of 20.65% (calcd 20.40%). The final product of degradation is Pd contaminated with a carbon atom.

**Table 4:** Thermal analysis results (TG and DTG) for AMI binary and ternary metal complexes

No.	Complexes	TG range (°C)	DTG_max_ (°C)	n*	Mass loss found (calcd)%	Total mass loss found (calcd)%	Assignment	Residues
1	[Pd(AMI)Cl_2_]	145–465	300, 324	2	36.95 (36.43)	57.60 (56.83)	2HCl, HCN	C + Pd
		465–730	550	1	20.65 (20.40)		C_2_H_4_N_2_	
2	[Cu(AMI)L^1^]	120–370	239	1	42.25 (42.25)	71.27 (71.22)	2CO_2_, NH_3_	2C + CuO
		370–1000	439, 770	2	29.02 (28.97)		C_2_H_4_N_2_O	
3	[Cu(AMI)L^2^·2H_2_O]	105–395	128, 238	2	26.43 (26.81)	69.44 (69.36)	CO_2_, 2H_2_O	C + CuO
		395–1000	438, 976	2	43.01 (42.55)		C_5_H_9_N_3_O

The first stage of complex 2 decompositions occurred at 120–370 °C and corresponded to the loss of hydrated NH3 and 2CO_2_ gas with a gradual mass loss of 42.25% (calcd 42.25%). The second and third decomposition stages at 370–1000 °C reflect C_2_H_4_N_2_O molecule loss with a mass loss of 29.02% (calcd 28.97%). The final product, CuO, was contaminated with carbon atoms.

Complex three's thermal degradation occurred in four degradation steps at 105–1000 °C. The first two degradation stages, at 105–395 °C, correspond to the loss of two coordinated water molecules and CO_2_ gas with a mass loss of 26.43% (calcd 26.81). The third and fourth degradation stages occurred at 395–1000 °C and reflected the loss of the C_5_H_9_N_3_O fragment with a mass loss of 43.01% (calcd 42.55%), leaving CuO as a residue with one carbon atom [66, 106].

### Anticancer studies

**Fig. 10:**
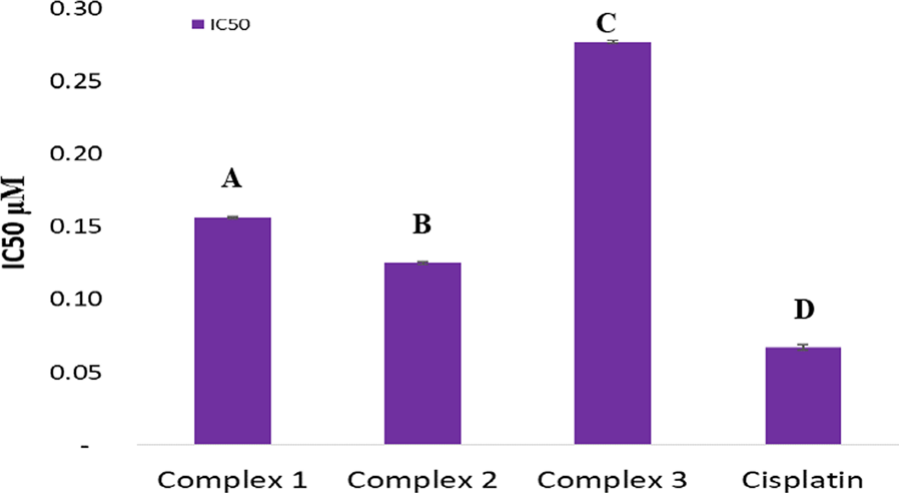
The graphical representation of the synthesized complexes' IC_50_ values in MCF-7 cancer cell lines. Different symbols indicate significant (*p* < 0.05) different means

**Fig. 11:**
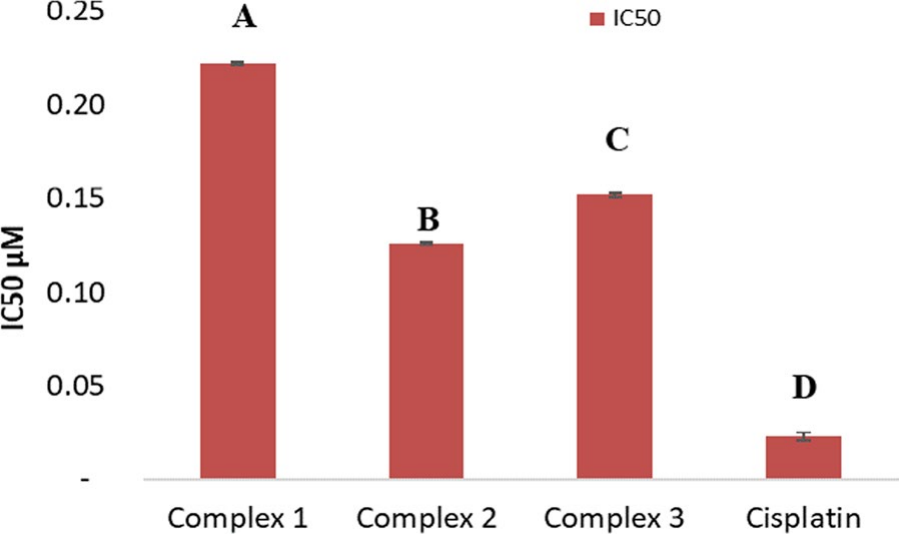
The graphical representation of the synthesized complexes' IC_50_ values in HeLa cancer cell lines. Different symbols indicate significant (*p* < 0.05) different means

**Fig. 12:**
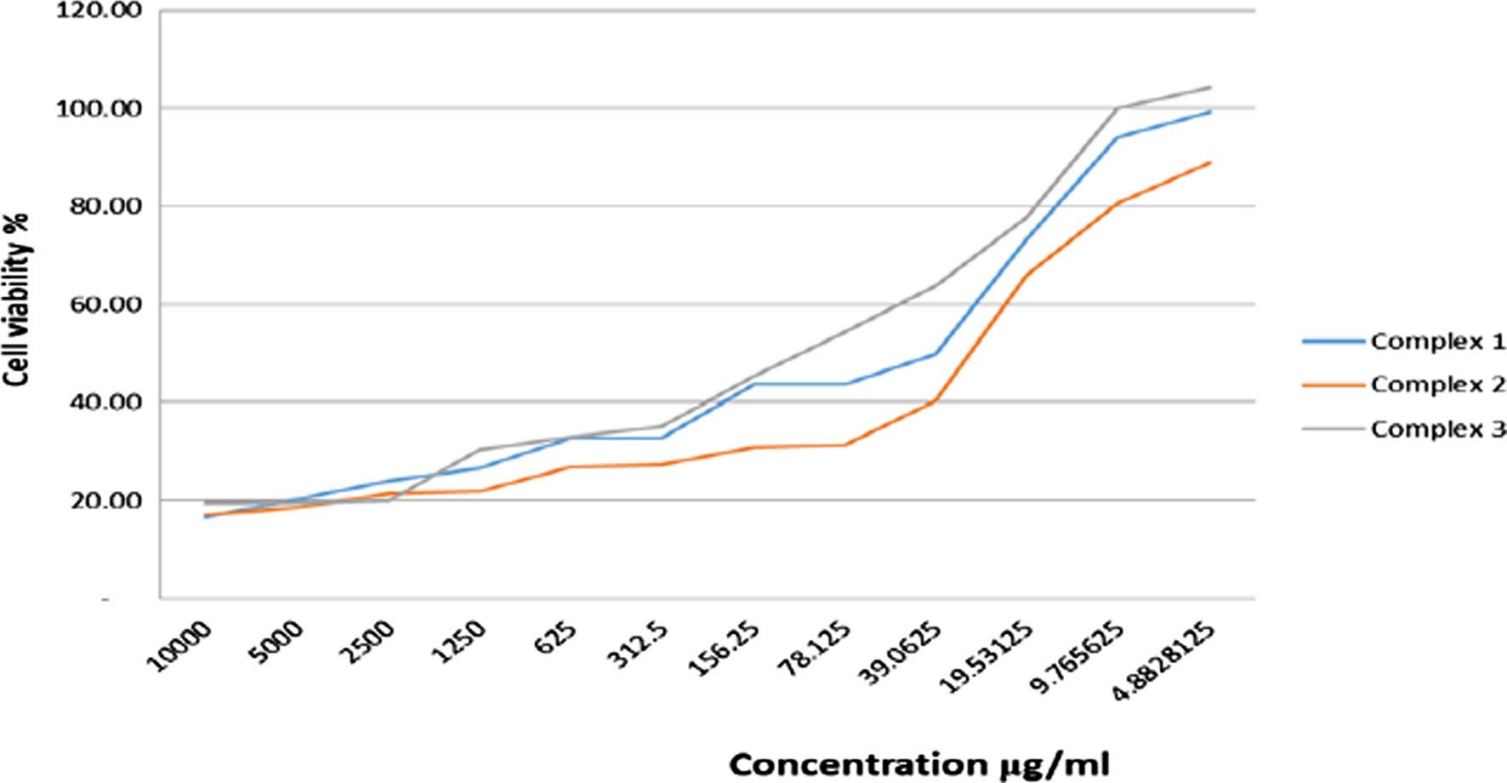
The survival curve of MCF-7 cells treated with different concentrations of synthesized complexes

**Fig. 13:**
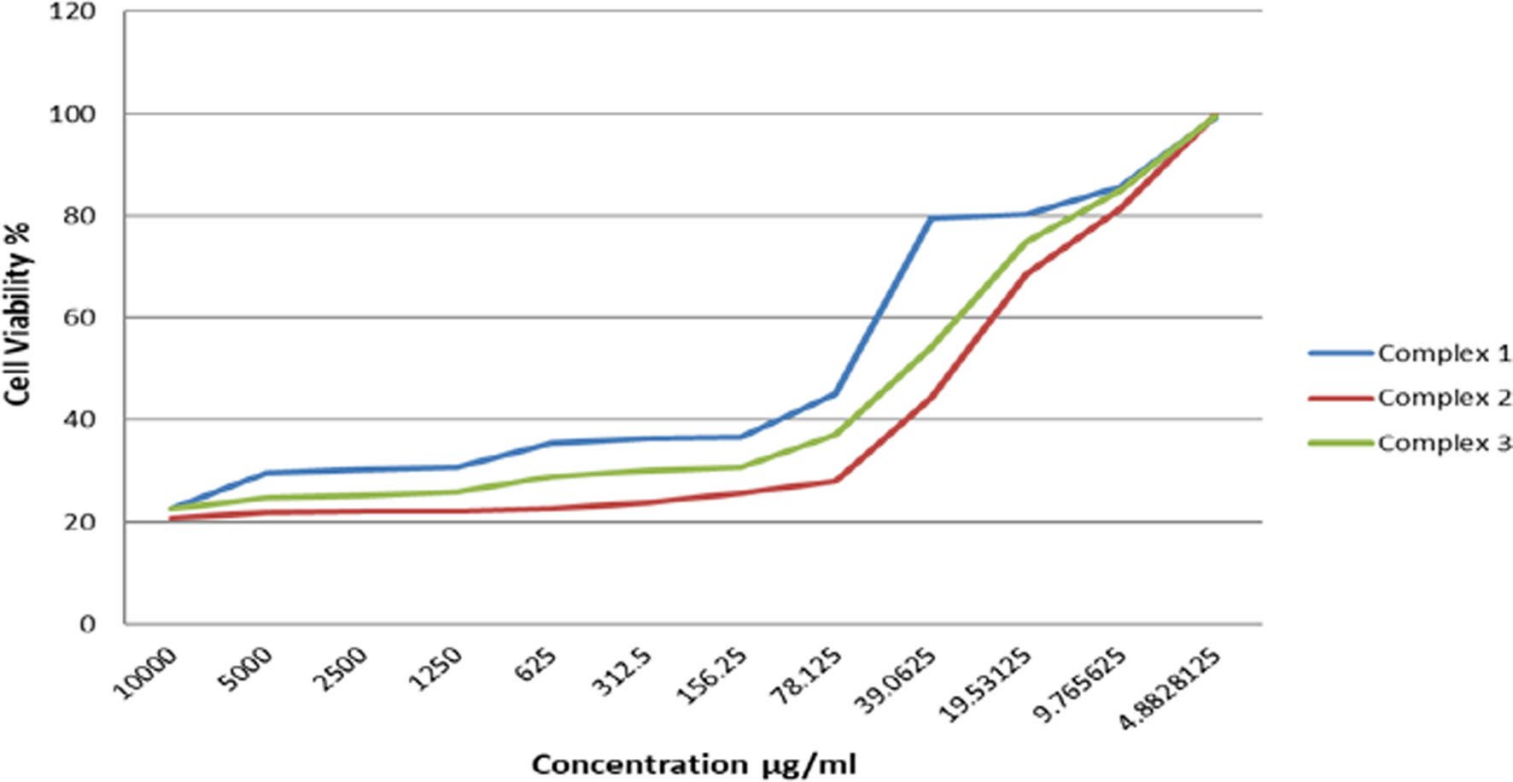
The survival curve of Hela cells treated with different concentrations of synthesized complexes

MCF-7 and HeLa cancer cells were incubated for 48 h with the newly prepared AMI complexes and cisplatin. IC_50_ values by MTT assay reflect cytotoxicity, and IC_50_ is defined as the concentration that can inhibit cancer cell growth by 50% [65] (Figs. 10, 11, 12, 13) and (Table 5). The standard drug (cisplatin) is strongly cytotoxic in MCF-7 and HeLa cells with IC_50_ values of 0.067 ± 0.0008 and 0.023 ± 0.002 μM. AMI complexes showed anticancer activity; however, their activity was less than cisplatin. Complexes one and two were more potent cytotoxic effects than complex three. Complex three was weakly cytotoxic against MCF-7 cancer cells, IC_50_ of 0.277 ± 0.002 μM. Complex two as the most active versus MCF-7 cells (IC_50_ = 0.125 ± 0.001 μM). The IC_50_ for the complex one was 0.156 ± 0.0006 μM. IC_50_s in HeLa cells for complex one, two, and three were 0.222 ± 0.0005, 0.126 ± 0.0009, 0.152 ± 0.001 μM, respectively. Complexes one and three were deemed to show moderate anticancer activity compared to complex two. Complex two as the most active against HeLa cells (IC_50_ = 0.126 ± 0.0009 μM). From the MTT results, it can be suggested that complex two (Cu(AMI)L^1^) shows strong anticancer activity against MCF-7 cells with IC_50_ 0.125 ± 0.001 μM, and its activity is close to cisplatin with IC_50_ 0.067 ± 0.0008 μM. Cu(AMI)L^1^ complex might be a potential pharmacological drug for breast cancer treatment. Cu(AMI)L^1^ complex's potential cytotoxicity can be linked to the strong DNA-complex binding ability [107, 108]. DNA-complex binding ultimately causes cell death [109]. Moreover, Tweedy's chelation theory suggests that the cytotoxic efficacy of complexes may be attributable to the focal metal atom [110]. Changes in coordination sites and type of metal ion affect biological activity via DNA binding ability [111]. We conclude that palladium chelate produces a strong anticancer activity in MCF-7 cells. Similarly, Karami et al. [112] found that palladium complexes show cytotoxicity toward multiple cancer cells, including MCF-7 breast cancer cells. Complex two was also more cytotoxic than the other metal complexes against HeLa cells. Thus, complex two is the most active against both MCF-7 and HeLa cells, and its toxicity is similar in these cell lines. The complexes, [Cr(MSEB)*Cl*_2_(H_2_O)_2_] (1), [Fe(MSEB)(NO_3_)_2_(H_2_O)_2_] (2) and [Cu(MSEB)*Cl*(H_2_O)_3_] (3) where MSEB = 1-(2-hydroxyethyl)-4,5-diphenyl-1H-imidazole-2-yl)(4-bromophenol)) were synthesized by Abu-Dief et al. [61]. Cytotoxicity of complexes was evaluated in vitro against Colon (HCT-116), Breast (MCF-7), and hepatic cellular (HepG-2) carcinoma cell lines. Results showed that complex 3 (MSEBCu) demonstrated the highest anticancer activity compared to other complexes to inhibit the growth of all cancer cells.

**Table 5:** IC_50_ values of synthesized complexes in μM in vitro

Complexes	MCF-7 cells	HeLa cells
	IC_50_ μM	IC_50_ μM
	Mean ± S.E.M.	Mean ± S.E.M.
Complex 1	0.156 ± 0.0006	0.222 ± 0.0005
Complex 2	0.125 ± 0.001	0.126 ± 0.0009
Complex 3	0.277 ± 0.002	0.152 ± 0.001
Cisplatin	0.067 ± 0.0008	0.023 ± 0.002

### Effect of complexes on oxidative stress in vitro

Malondialdehyde (MDA) level and catalase activity as oxidative and antioxidative stress markers were measured in MCF-7 and HeLa cells treated with metal complexes compared to untreated cancer cells. Cancer cells were treated for 48 h with complex one, two, and three at IC_50_ concentrations for MCF-7 cells of 0.156, 0.125, 0.277 μM, respectively, and 0.222, 0.126, 0.152 μM, respectively, for HeLa cells.

#### Effect of complexes on catalase activity

**Fig. 14:**
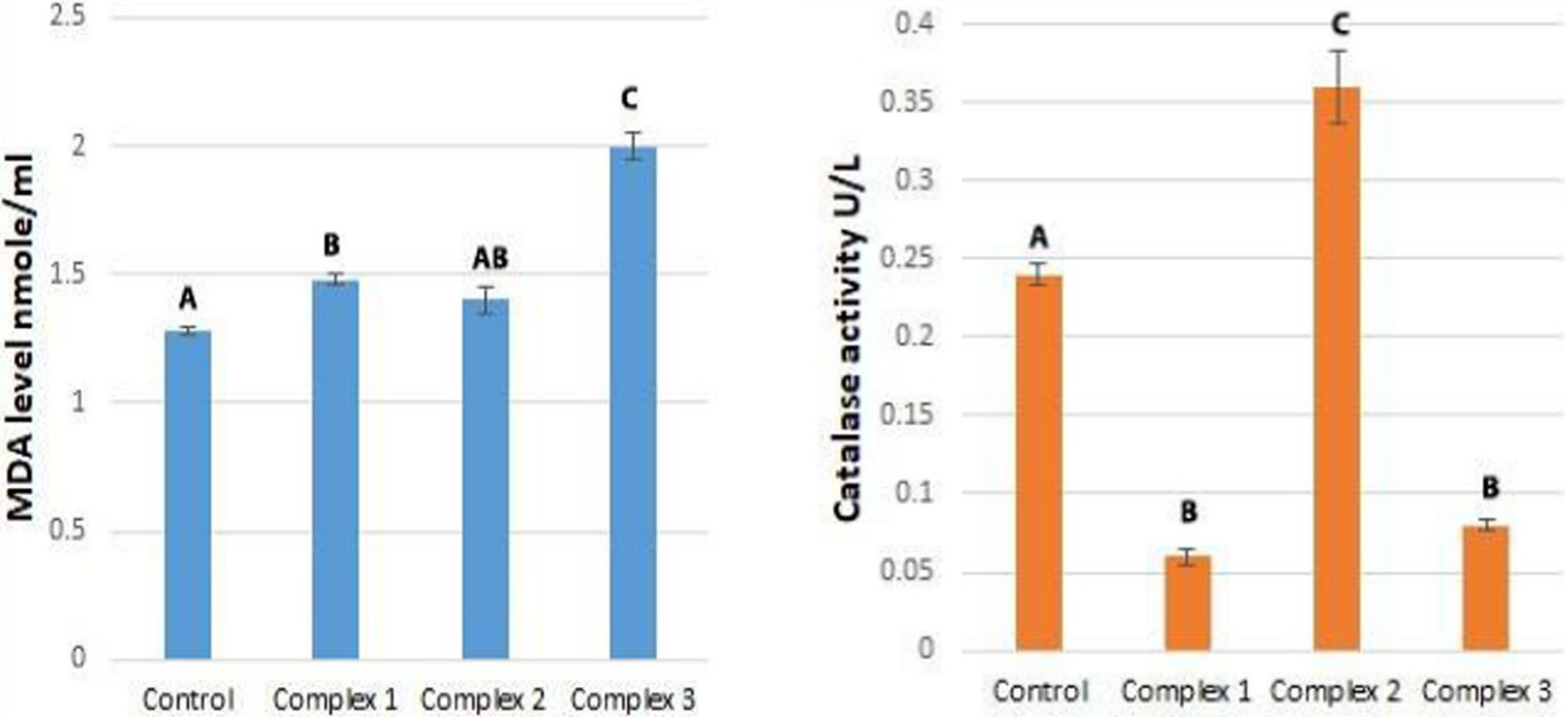
The graphical representation of the effect of complexes on some oxidative stress parameters in the MCF-7 cells. Different symbols indicate significant (*p* < 0.05) different means

**Fig. 15:**
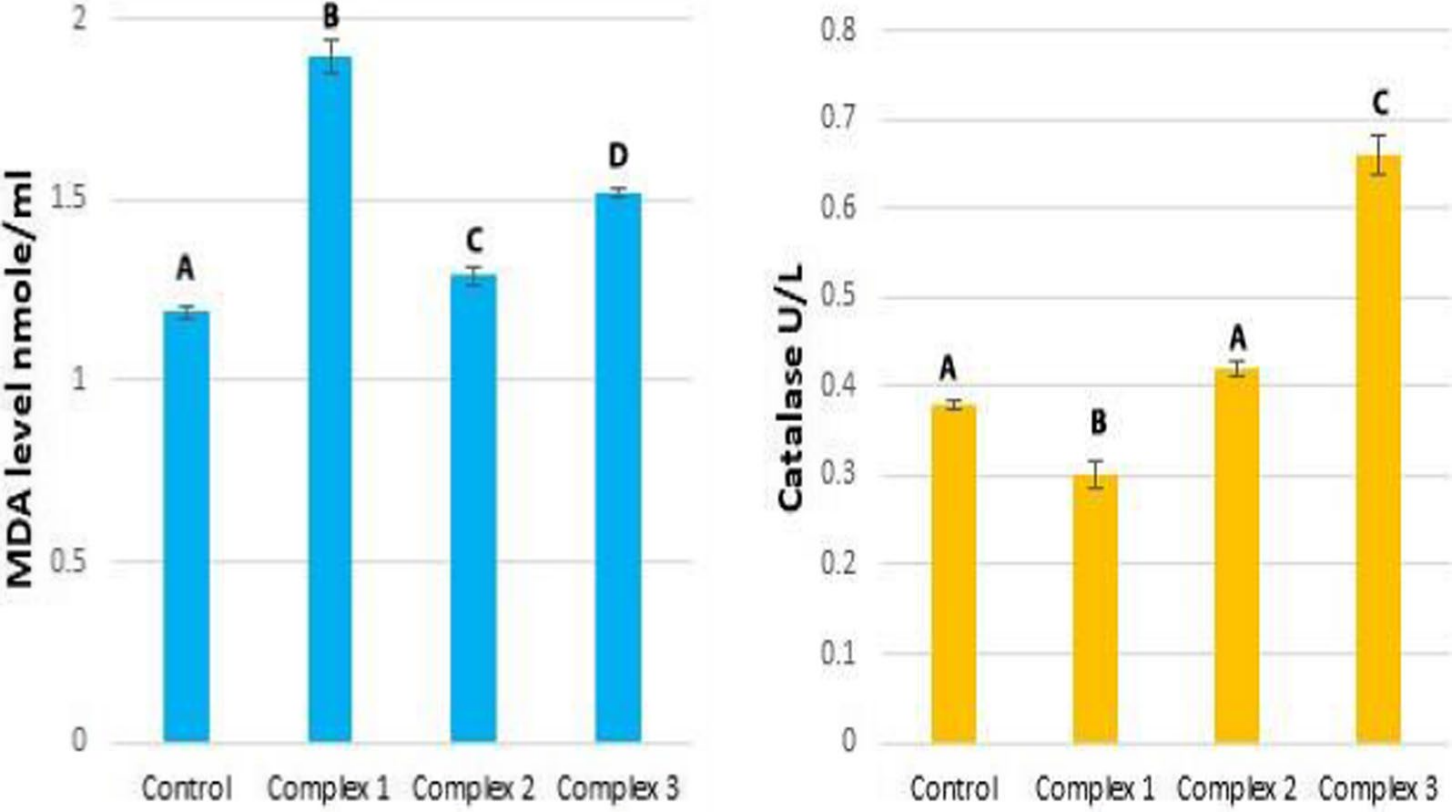
The graphical representation of the effect of complexes on some oxidative stress parameters in the HeLa cells. Different symbols indicate significant (*p* < 0.05) different means

MCF-7 cells treated with complex two induced a significant increase in catalase activity (50%) in comparison to the untreated cells (control) and other complexes one and three (− 66.7% and − 75%, respectively (Table 6 and Fig. 14). Compared to the control group, cells treated with complexes one and three showed a significant decline in catalase activity. Also, non-significant changes in catalase activity were observed after treating cells with complexes one and two. HeLa cells treated with complex three displayed significantly increased catalase activity (73.7%) in comparison with control and other complexes one and two (10.5% and − 21.1%, respectively) (Table 7 and Fig. 15). In addition, cells treated with complex two showed significantly increased levels of catalase activity in comparison to cells treated with a complex one. Also, the catalase activity of cells treated with complex two is not significantly increased compared to the control. Cells treated with complex one showed significantly reduced catalase activity compared to control cells. MCF-7 and HeLa cells responded differently to a treatment. MCF-7 cells treated with complex two showed increased catalase levels, but complexes one and three decreased catalase activity compared to untreated cells. In contrast, complex one (palladium complex) decreased catalase activity in HeLa cells, and complexes two and three (copper complexes) increased activity compared to untreated cells. Responses in catalase activity appear to depend on the type of cancer cell.

**Table 6:** Effect of complexes on some oxidative stress parameters in MCF-7 cells

Oxidative stress parameters	Control group mean ± S.E.M.	Treated groups mean ± S.E.M.					
		Complex 1		Complex 2		Complex 3	
		Value	% D	Value	% D	Value	% D
MDA (nmol/mL)	1.28 ± 0.015	1.48 ± 0.02	15.6%	1.40 ± 0.05	9.4%	2 ± 0.055	56.25%
	A	B		AB		C	
Catalase (U/L)	0.24 ± 0.007	0.06 ± 0.005	− 75%	0.36 ± 0.023	50%	0.08 ± 0.003	− 66.7%
	A	B		C		B	

Different symbols indicate significant (*p* < 0.05) different means

**Table 7:** Effect of complexes on some oxidative stress parameters in the HeLa cells

Oxidative stress parameters	Control group mean ± S.E.M.	Treated group mean ± S.E.M.					
		Complex 1		Complex 2		Complex 3	
		Value	% D	Value	% D	Value	% D
MDA (nmole/mL)	1.19 ± 0.017	1.90 ± 0.05	59.7%	1.29 ± 0.023	8.4%	1.52 ± 0.01	27.7%
	A	B		C		D	
Catalase (U/L)	0.38 ± 0.005	0.30 ± 0.015	− 21.1%	0.42 ± 0.01	10.5%	0.66 ± 0.02	73.7%
	A	B		A		C	

Different symbols indicate significant (*p* < 0.05) different means

Catalase is a crucial antioxidant enzyme in cellular defense against oxidative stress, as shown in various cancers [113]. Catalase converts H_2_O_2_ to water and molecular oxygen [114], thus protecting cells from H_2_O_2_-induced stress [115]. Catalase is thus an essential index for determining oxidative stress levels [116, 117]. Some complex-treated cells exhibit low catalase activity, promoting oxidative stress by promoting mitochondrial dysfunction, elevated ROS levels, and reduced antioxidant defenses [118]. Treatment of HeLa cells with copper complexes and MCF-7 cells with complex two (copper complex) induces an increase in catalase activity that may strengthen protection from oxidative damage by scavenging excess free radicals ‏ [119]. Also, treatment of MCF-7 and HeLa cells with the palladium chelate caused a decrease in catalase activity.

#### Effect of complexes on malondialdehyde (MDA) levels

Treatment with complex three caused a significant increase in MDA levels in MCF-7 cells (56.25%) compared with untreated cells and complexes one and two (15.6% and 9.4%, respectively) (Table 6 and Fig. 14). A non-significant difference was seen between cells treated with complexes one versus two in MDA levels. No significant change was observed in MDA levels between cells treated with complex two and untreated cells. Further, complex one caused a significant increase in MDA levels relative to untreated cells.

MDA levels in HeLa cells treated with complex one increased significantly (59.7%) compared with untreated cells and complexes two and three (8.4% and 27.7%, respectively) (Table 7 and Fig. 15). MDA levels increased significantly in cells treated with complex three compared to complex two and untreated cells. The increase in MDA levels after the complex two treatment was significant. In response to oxidative stress, reactive intermediates can alter membrane bilayers and cause polyunsaturated fatty acid lipid peroxidation [120–122]. Reactive aldehydes such as 4-hydroxynonenal (4-HNE) and Malondialdehyde (MDA) are generated during lipid peroxidation [123, 124]. A significant difference in MDA levels between cancer cell types (MCF-7 and HeLa) after complex treatment was noted.

Nevertheless, MCF-7 cells treated with complex two produced a non-significant increase in MDA levels compared with untreated cells. MDA levels increased in MCF-7 cells treated with complex three (copper complex) as compared to untreated cells; similar results were reported by Khan et al. [125] and Alharbi et al. [126]. The highest MDA levels were seen after treatment with the copper complex. These levels may be related to reactive oxygen species (ROS) generation in carcinoma cells. An elevated ROS level causes lipid-based radical formation and accelerates MDA production [127]. HeLa cells treated with complex one (pallidum complex) showed elevated MDA levels relative to untreated cells and the copper complexes. Thus, the type of metal and ligand influenced the levels of MDA. Metals can induce lipid peroxidation, such as copper [128]. MDA levels in cancer cells depend on the concentration of complexes; higher concentrations lead to higher MDA levels. For example, the treatment of MCF-7 cells with complex three at a higher concentration than other complexes (0.277 μM) and treatment of HeLa cells with complex one at a concentration (0.222 μM). Hence, complexes that exhibited low cytotoxicity caused an increase in MDA levels in these cells. Finally, cancer cells treated with complex three showed increased MDA levels in both cell types; complexes one and three induced an increase in MCF-7 cells. Complex one in HeLa cells caused a decrease in catalase activity and increased oxidative stress, as evidenced by elevated MDA levels and reduced catalase activity. Cell membranes, mitochondria, DNA, and nuclei are damaged by increased oxidative stress, resulting in cancer cell death via apoptosis [129–131].

### Assessment of antioxidant activity utilizing the DPPH radical scavenging method

**Fig. 16:**
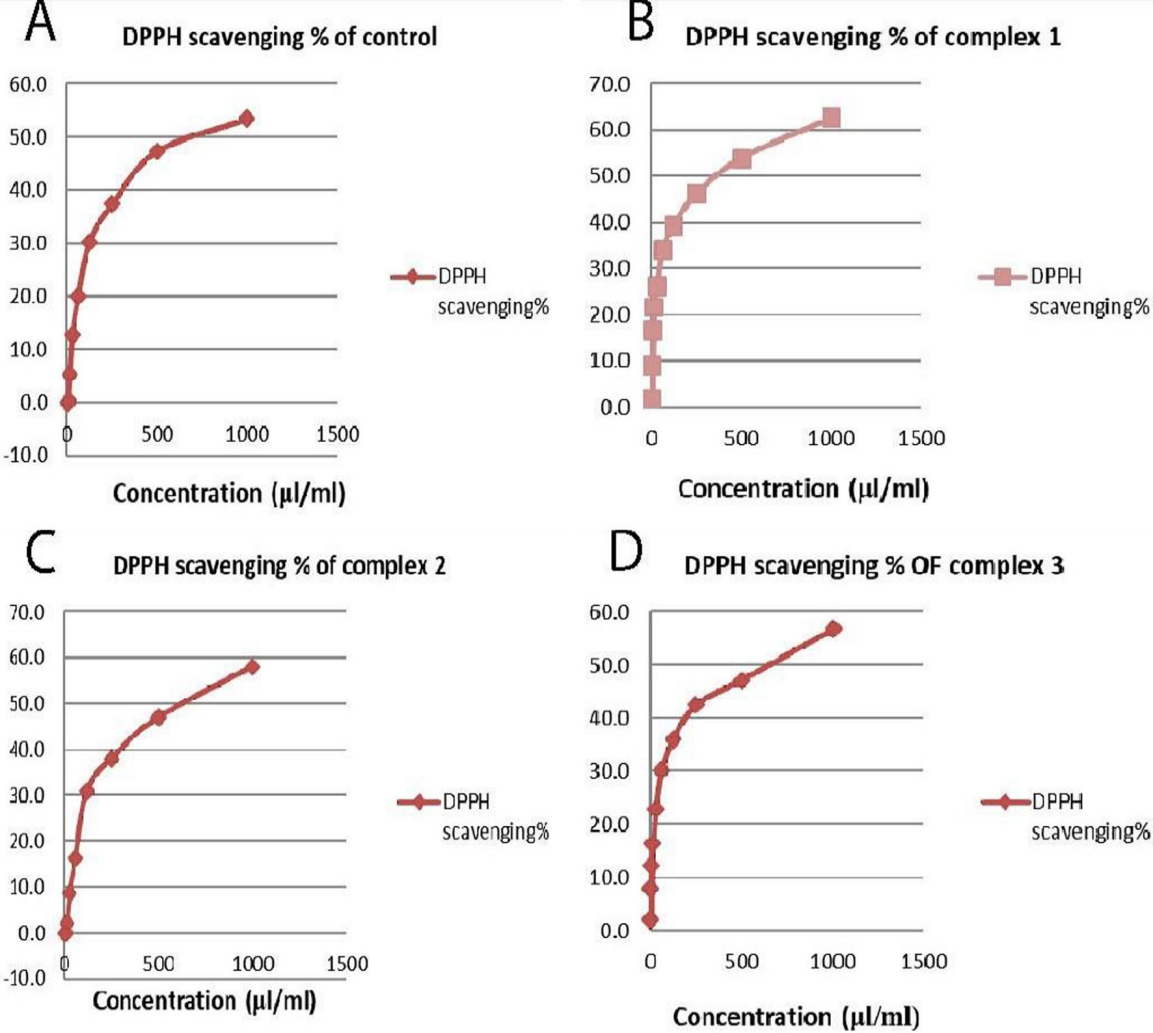
The complexes' free radical scavenging activity in MCF-7 cells in comparison to untreated cells (control): **A** DPPH scavenging% of control, **B** DPPH scavenging% of complex one, **C** DPPH scavenging% of complex two, **D** DPPH scavenging% of complex three

**Fig. 17:**
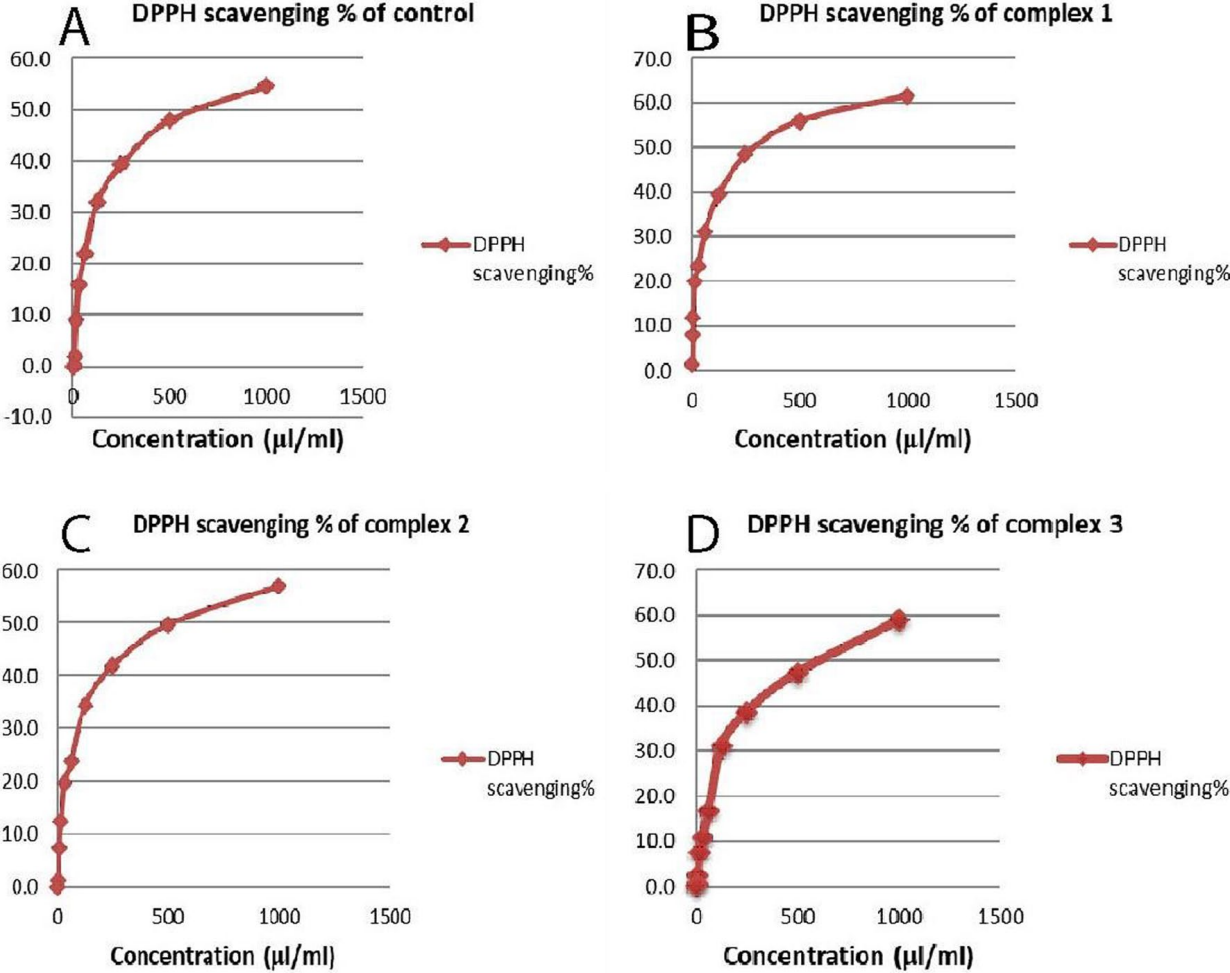
The complexes' free radical scavenging activity in the HeLa cells in comparison to untreated cells (control): **A** DPPH scavenging% of control, **B** DPPH scavenging% of complex one, **C** DPPH scavenging% of complex two, **D** DPPH scavenging% of complex three

The newly prepared AMI complexes' antioxidant activity on MCF-7 and HeLa cancer cell lines is assessed using a DPPH (1,1-diphenyl-2picrylhydrazyl) radical scavenging test. MCF-7 cells were treated for 48 h with complexes one, two, and three at their IC_50_ values of 0.156, 0.125, and 0.277 μM, respectively. Likewise, HeLa cells were treated at their IC_50_ values of 0.222, 0.126, 0.152 μM, respectively. Radical scavenging activity increased with complex concentrations (Figs. 16, 17). SC_50_ values in MCF-7 cells were 227.5 ± 0.28, 901.6 ± 0.23, and 612.5 ± 0.57 μL/mL, respectively; the SC_50_ was 992 ± 1.15 μL/mL in untreated cells. Similarly, HeLa cell SC_50_'s were 361 ± 1.2, 619.5 ± 0.86, and 835.5 ± 0.63 μL/mL, respectively, compared to 814.5 ± 1.45 μL/mL in untreated cells. Better DPPH radical scavenging efficacy is indicated by lower SC_50_ values [132]. Complex one in MCF-7 cells exhibits higher scavenging activity than other complexes and control cells. This complex may be most effective in scavenging free radicals.

Similarly, treating the HeLa cells with complex one produced the lowest SC_50_, again suggesting relatively efficient free radical scavenging. Complex one might be a better candidate drug where free radical generation is a primary concern. Complexes two and three might be less useful with more moderate activity. Complex antioxidant activity can be attributed to the electron-withdrawing properties of metal ions, which facilitate the release of H + atoms to reduce the DPPH radical [133]. [Pd(1,2-dimethylimidazole)_2_Cl_2_] (1) and [Pd(benzimidazole)_2_Cl_2_] (2) were synthesized by Sadaf et al. [134]. A Free radical scavenging assay assessed the antioxidant activity. Scavenging assay results showed that complex 1 demonstrated the most potent scavenging activity.

### Evaluation of DNA damage by comet assay

#### Effect of newly prepared AMI complexes on DNA

**Fig. 18:**
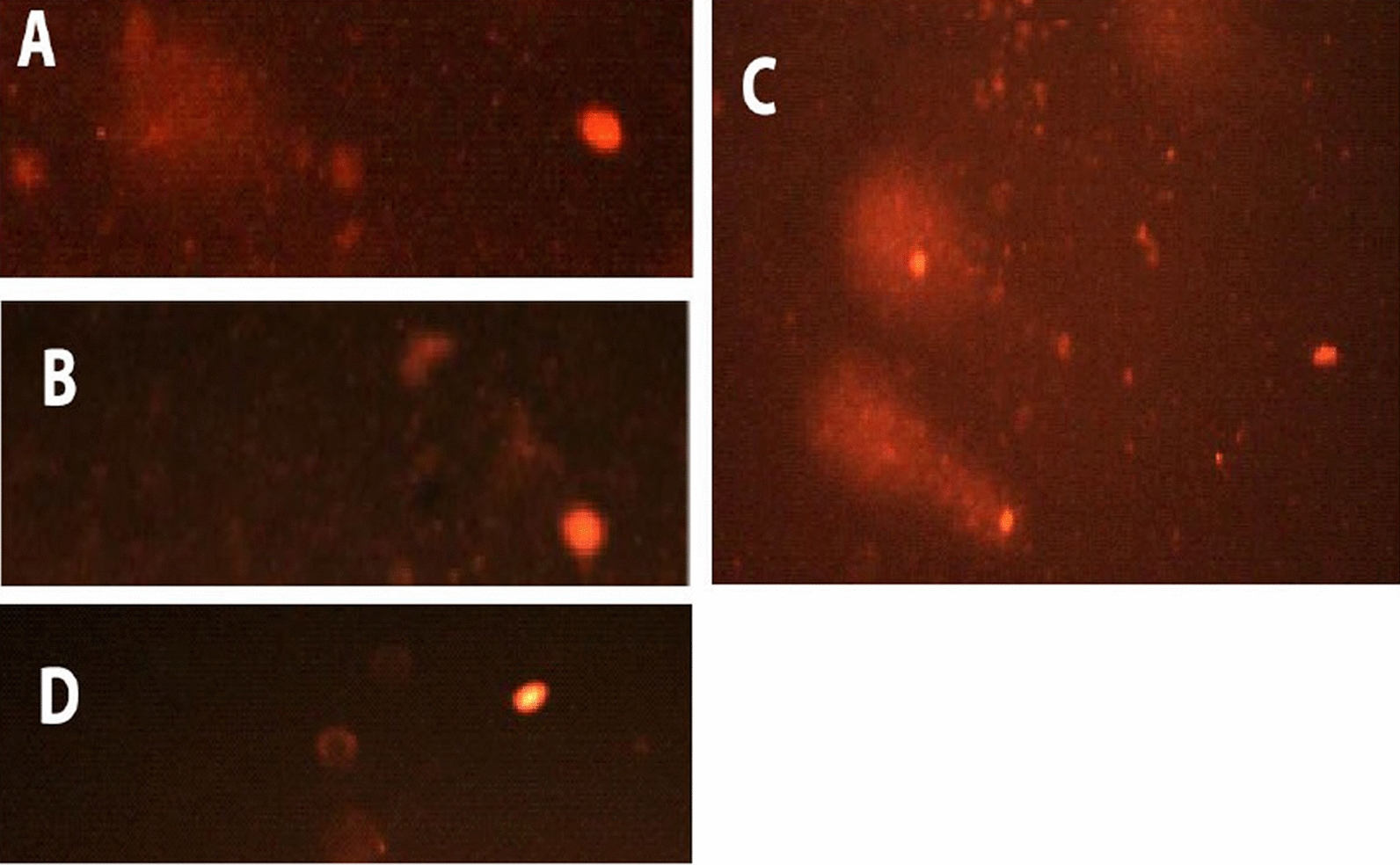
Fluorescence-stained comet images of MCF-7 cells. **A** Control, **B** Comet image of MCF-7 treated with complex one with IC_50_ 0.156 Μm, **C** Comet image of MCF-7 treated with complex two with IC_50_ 0.125 μM, **D** Comet image of MCF-7 treated with complex three with IC_50_ 0.277 μM

The comet assay assesses three distinct parameters: tail length, percentage of DNA in the tail, and tail moment. These parameters reflect DNA damage caused by newly prepared AMI complexes. MCF-7 cells were treated for 48 h with complexes one, two, and three at their IC_50_ values of 0.156, 0.125, and 0.277 μM, respectively (Table 8 and Figs. 18, 20). Damage percentage increased significantly when cells were treated with complex two compared to control and other complexes. Also, complex three significantly increased damage percentage compared to complex one and control cells. Complex one produced only a non-significant increase in damage percentage compared to control cells. In particular, complex two induce significant increases in tail length and tail moment compared to other complexes and control cells.

**Table 8:** Effect of complexes (IC_50_ = 0.156, 0.125, 0.277 μM, respectively) on the DNA of MCF-7 cells: Comet Damage (%), tail length, DNA in the tail (%), and tail moment

Parameters	Control group mean ± S.E.M.	Treated group mean ± S.E.M.					
		Complex 1		Complex 2		Complex 3	
		Value	% D	Value	% D	Value	% D
Comet (damage) (%)	10.5 ± 0.29	10.5 ± 0.35	0%	18 ± 0.17	71.4%	15 ± 0.06	42.8%
	A	A		B		C	
Tail length (μm)	7.8 ± 0.1	7.3 ± 0.02	− 6.4%	9.33 ± 0.04	27.8%	6.5 ± 0.01	− 16.6%
	A	B		C		D	
DNA in tail (%)	8.3 ± 0.05	7.6 ± 0.09	− 8.4%	7.5 ± 0.07	− 9.6%	7.5 ± 0.03	− 9.6%
	A	B		B		B	
Tail moment (μm)	0.74 ± 0.01	0.57 ± 0.005	− 22.9%	0.83 ± 0.02	12.1%	0.56 ± 0.008	− 24.3%
	A	B		C		B	

Different symbols indicate significant (*p* < 0.05) different means

In contrast, complexes one and three displayed significant decreases in tail length and tail moment compared to control cells. A non-significant difference in the tail moment between complexes one and three was also observed. After treatment with all three complexes, a significant decrease in the percent of DNA in the tail was seen. Also, no significant differences in the percent DNA in the tail were observed among complexes.

**Fig. 19:**
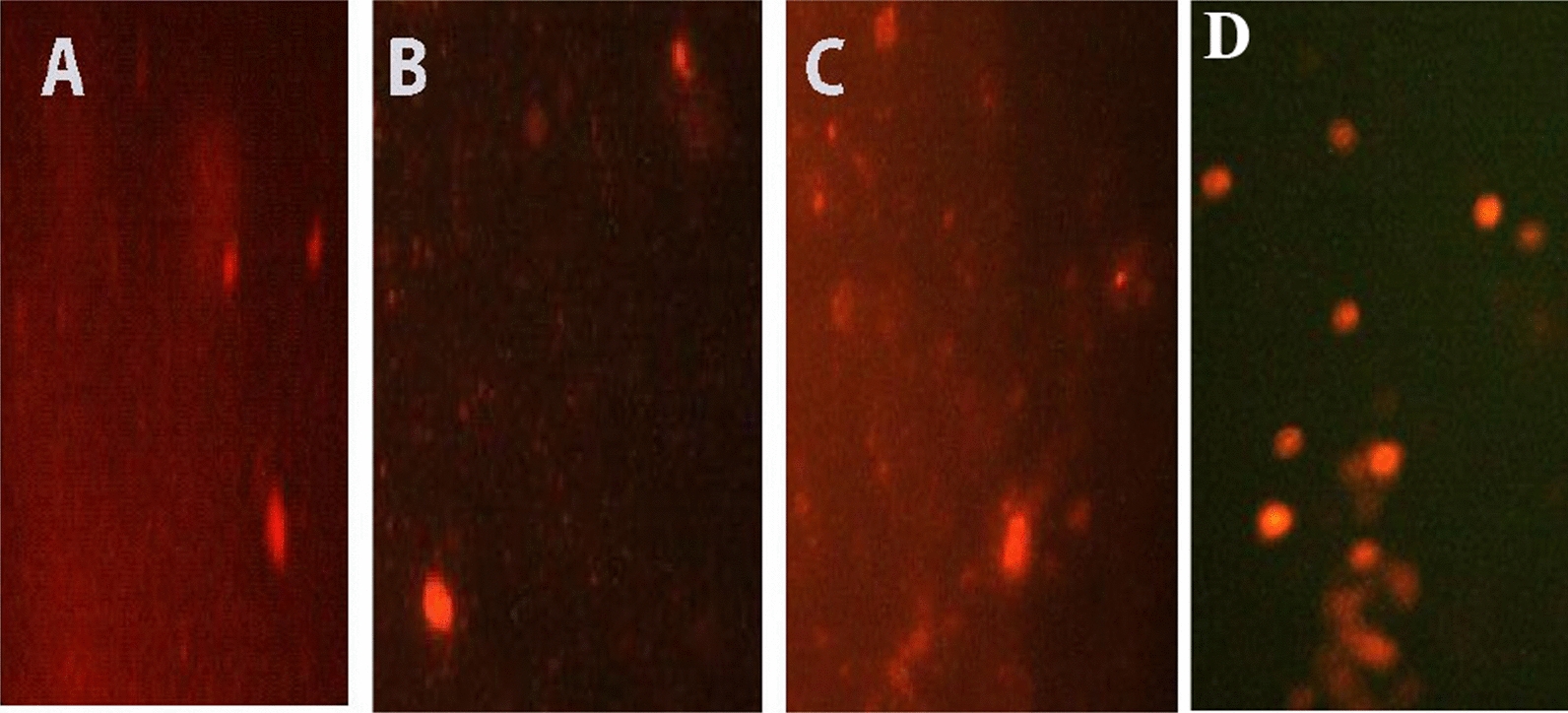
Fluorescence-stained comet images of HeLa cells. **A** Control, **B** Comet image of HeLa treated with complex one with IC_50_ 0.222 μM, **C** Comet image of HeLa treated with complex two with IC_50_ 0.126 μM, **D** Comet image of HeLa treated with complex three with IC_50_ 0.152 μM

**Fig. 20:**
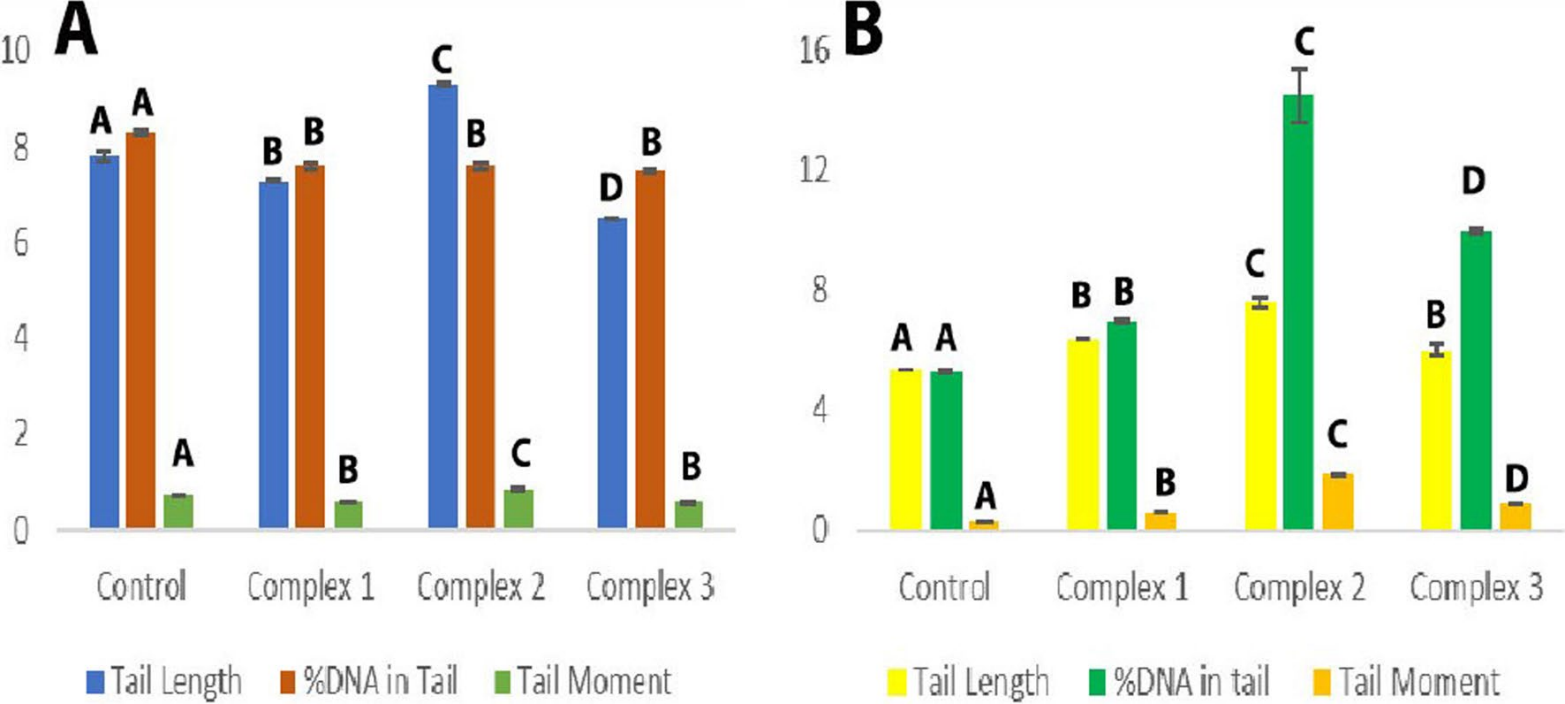
DNA damage in terms of Tail Length, %DNA in tail, and Tail Moment in **A** MCF-7, **B** HeLa cells. Different symbols indicate significant (*p* < 0.05) different means

HeLa cells were treated for 48 h with complexes one, two, and three at their IC_50_ values of 0.222, 0.126, 0.152 μM, respectively (Table 9 and Figs. 19, 20). Percent damage, tail length, tail moment, and percentage of DNA in the tail increase significantly compared to control cells. Complex one exhibited the greatest overall impact, significantly increasing the percent damage. Further, significant increases in the tail length, percent DNA in the tail, and tail moment were induced by complex two; other complexes showed lesser effects. A significant increase in DNA in tail and tail moment was induced by complex three compared to complex one, and a non-significant difference in tail length between complexes one and three were noted. The tail length, percent DNA in the tail, and the tail moment significantly increased after complex one treatment.

**Table 9:** Effect of complexes (IC_50_ = 0.222, 0.126, 0.152 μM, respectively) on the DNA of HeLa cells: Comet Damage (%), tail length, DNA in Tail (%), and tail moment

Parameters	Control group mean ± S.E.M.	Treated group mean ± S.E.M.					
		Complex 1		Complex 2		Complex 3	
		Value	% D	Value	% D	Value	% D
Comet (damage) (%)	8.6 ± 0.08	18.8 ± 0.12	118.6%	11.3 ± 0.23	31.4%	12 ± 0.4	39.5%
	A	B		C		D	
Tail length (μm)	5.37 ± 0.02	6.33 ± 0.005	17.9%	7.6 ± 0.17	41.5%	6 ± 0.2	11.7%
	A	B		C		B	
DNA in tail (%)	5.32 ± 0.01	6.99 ± 0.05	31.2%	14.5 ± 0.86	172.5%	9.96 ± 0.09	87.2%
	A	B		C		D	
Tail moment (μm)	0.27 ± 0.006	0.58 ± 0.003	114.8%	1.89 ± 0.04	600%	0.91 ± 0.01	237%
	A	B		C		D	

Different symbols indicate significant (*p* < 0.05) different means

The alkaline comet test was used in the current study. With the lowest IC50 in MCF cells, Complex Two exhibited significantly greater DNA damage than other complexes for all three parameters-tail length, percent DNA in the tail, and tail moment. Treatment of MCF-7 cells with complexes one and three showed intact DNA (the head), similar to control cells. These complexes did not induce significant DNA damage. Significant decreases in comet parameters of tail length, percent DNA in the tail, and tail moment were noted.

HeLa cells treated with complex two, with the lowest IC_50_, induced greater DNA damage than other complexes. Also, complex three showed greater DNA damage than complex one. Still, complex one did cause significant damage. Thus, HeLa cells treated with copper complexes (complexes two and three, whose IC_50_ is lower than the palladium complex) induced significantly more DNA damage than HeLa cells treated with the palladium complex (complex one). Serious DNA damage, as evidenced by a significant increase in comet assay parameters as tail length, %DNA in the tail, and tail moment in the complexes-treated HeLa cells versus the control cells. Complex two induces the greatest DNA damage and similarly affects HeLa and MCF-7 cells. DNA fragmentation in HeLa cells treated with any complex and MCF-7 cells treated with complex two displayed the comet-like form. No DNA fragmentation was observed in control cells where no “comet” formed. Electrophoretic migration of DNA fragments increased toward the anode compared to control cells. This was observed from comet parameters and the figures of comets (Figs. 18c and 19b–d). ROS mediates DNA strand breaks caused by complexes, such as superoxide anions and hydroxyl radicals [135].

### DNA fragmentation by gel electrophoresis

**Fig. 21:**
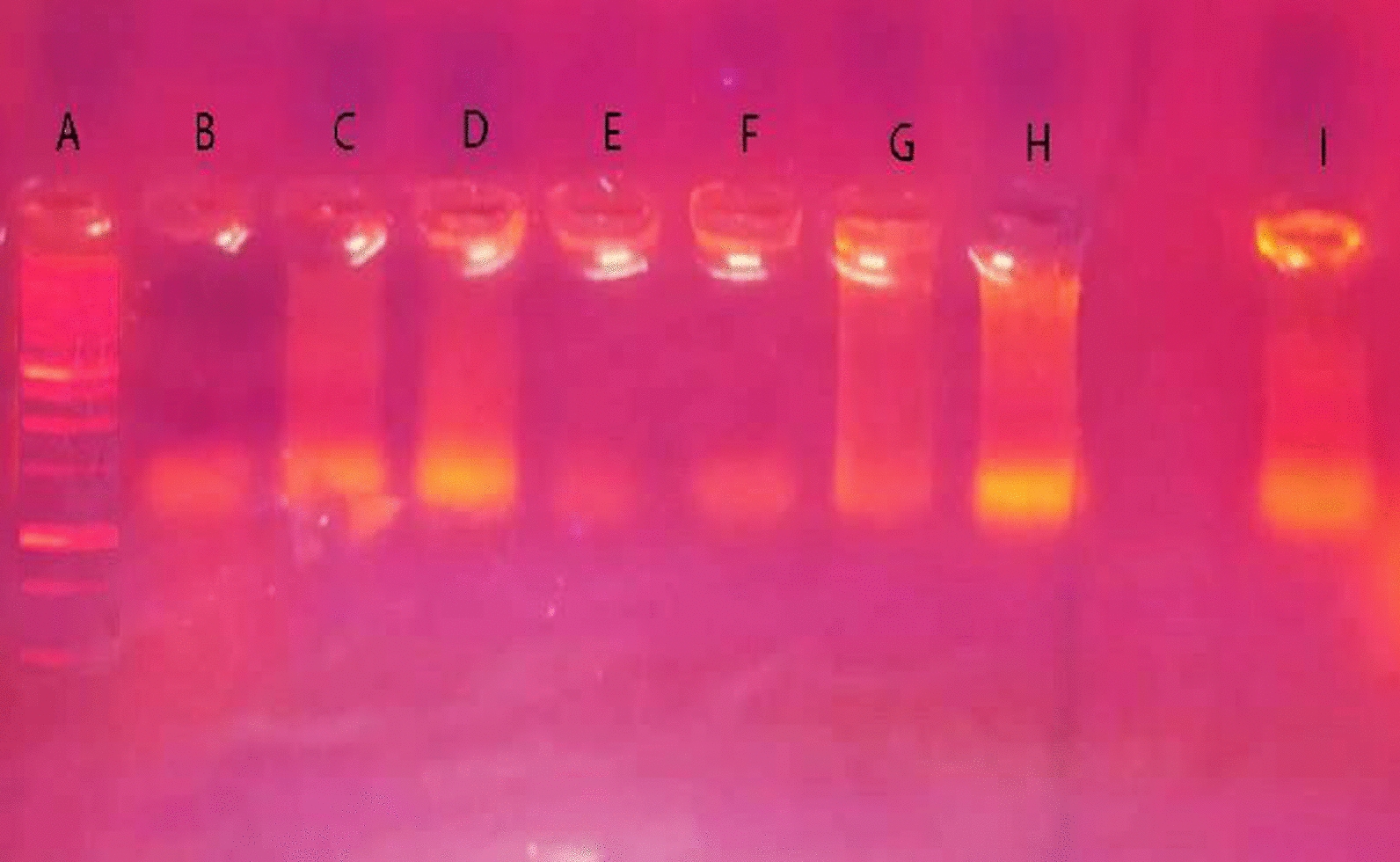
Study of DNA degradation of cancer cells treated with complexes using agarose gel electrophoresis. Lane A: DNA marker: Lane B: Untreated DNA of HeLa cells: Lane C: DNA + complex two (Cu(AMI)L^1^): Lane D: DNA + complex three (Cu(AMI)L^2^·2H_2_O): Lane E: DNA + Complex one (Pd(AMI)Cl_2_). For MCF-7 cells, Lane F: untreated DNA of MCF-7 cells: Lane G: DNA + complex two: Lane H: DNA + complex three: Lane I: DNA + complex one

DNA fragmentation of cancer cells treated with complexes compared to untreated was examined using agarose gel electrophoresis (Fig. 21). DNA from untreated MCF-7 cells (lane F) does not show fragmentation. However, DNA from cells treated with complexes (lanes G, H, and I) exhibits an unusual smearing pattern and fragmentation. DNA from cells treated with complex three (lane H) exhibits large, slow-moving fragments, as seen by the smear's increased intensity toward the lane's onset. DNA fragments in cells treated with complex one (lane I) are more mobile than fragments in lanes G and H, where the intensity of the smear is significant toward the end of the lane, indicating tiny fragments.

DNA of untreated HeLa cells (lane B) shows no smearing, confirming normal DNA. Noticeable smearing is seen for complexes two and three (lanes C and D). Conversely, treatment with complex one (lane E) induced no smearing. DNA fragments in lanes C and D are more mobile than untreated DNA (lane B) and lane E. Perhaps, DNA intensity from treated cells decreased because of the binding‏ of complexes to DNA [136]. Moreover, complex two has a comparable impact on HeLa and MCF-7 treated cells. Thus, metal ions play a crucial role in DNA interactions and may inhibit the growth of pathogens by interacting with the genome [137].

### Antimicrobial activity

#### Antibacterial activity

**Fig. 22:**
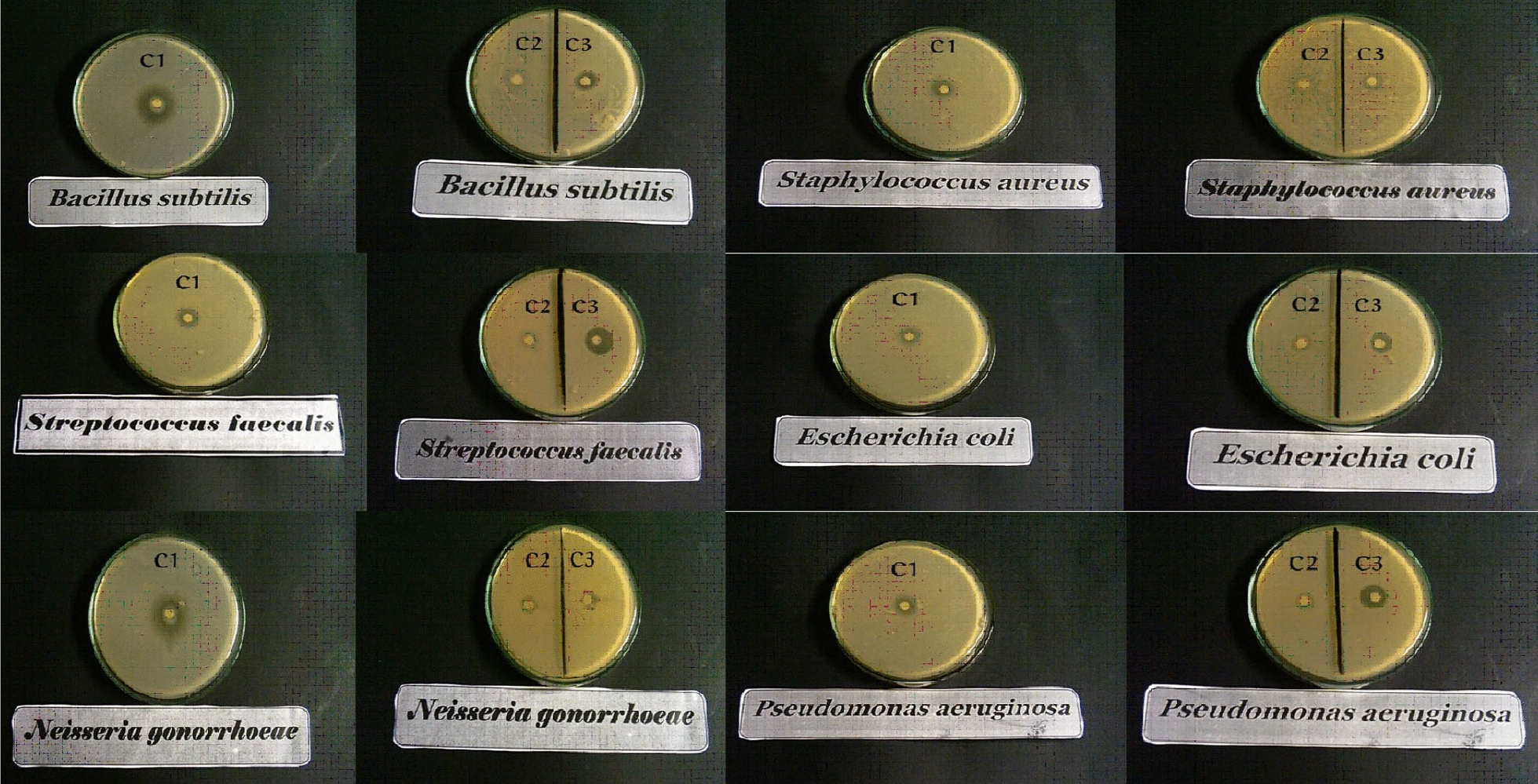
The antibacterial activity of complexes

**Fig. 23:**
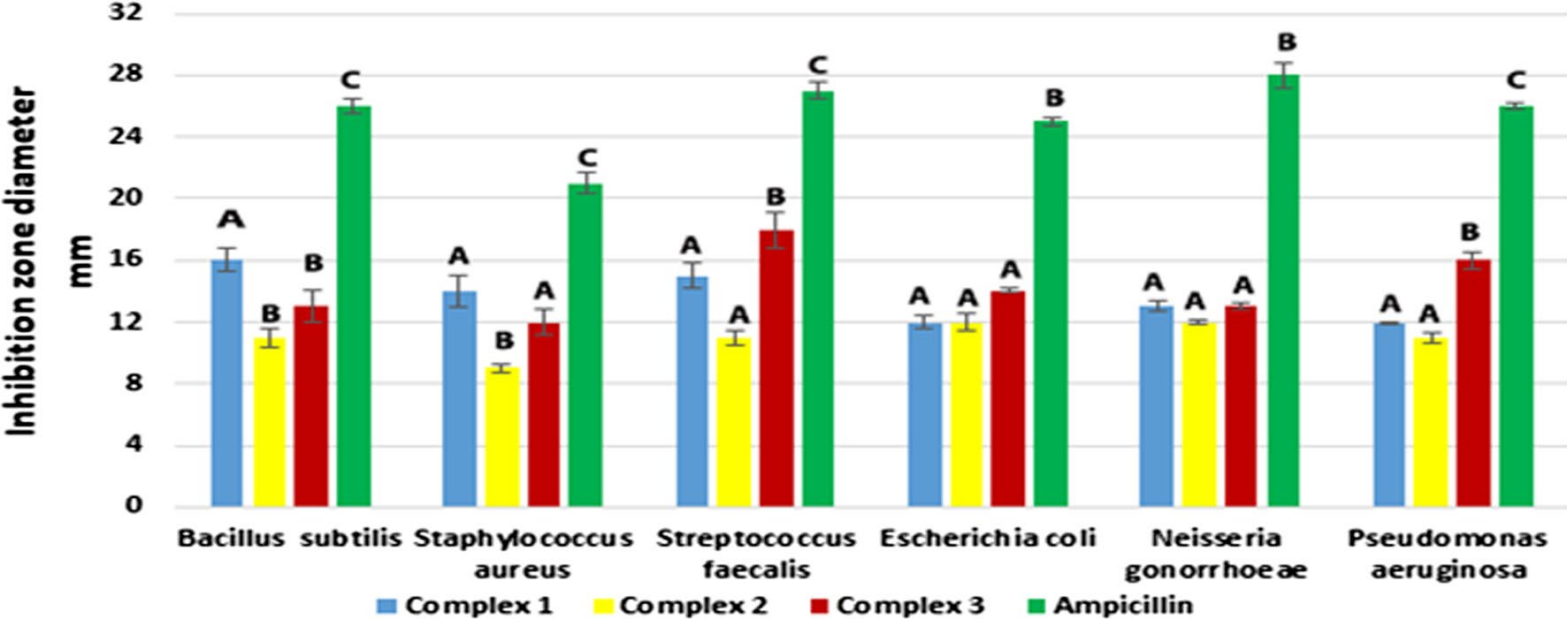
In vitro antibacterial activity of complexes regarding inhibition zone diameter in mm in vitro. Different symbols indicate significant (*p* < 0.05) different means

Antibacterial activity was assessed versus six bacterial strains using ampicillin as a reference drug and DMSO as the solvent. DMSO did not affect bacterial growth. Inhibition zones were measured in millimeters (see Figs. 22, 23). AMI complexes were less effective antibiotics than ampicillin. Results in Table 10. The complex one caused the largest inhibition zone (16 ± 0.76 mm) against *Bacillus subtilis*. This zone was more prominent than for complexes two and three (11 ± 0.57 mm and 13 ± 1.0, respectively)., Similarly, complex one showed greater activity versus *Staphylococcus aureus* than complex two (inhibition zones of 14 ± 1 and 9 ± 0.28 mm, respectively). The size of inhibition zones did not differ between complexes one and three. Complex three exhibited the largest inhibition zone against *Streptococcus faecalis* (18 ± 1.15 mm); significantly smaller zones were noted for complexes one and two (15 ± 0.87 and 11 ± 0.5 mm, respectively). Zones for the latter two complexes did not differ insignificantly.

**Table 10:** Inhibition zone diameter of complexes in mm in vitro

Complexes	Antimicrobial activity							
	(Inhibition zone diameter (mm)							
	Mean ± S.E.M.							
	Bacterial species						Fungal strains	
	G +			G −				
	*Bacillus subtilis*	*Staphylococcus aureus*	*Streptococcus faecalis*	*Escherichia coli*	*Neisseria gonorrhoeae*	*Pseudomonas aeruginosa*	*Aspergillus flavus*	*Candida albicans*
Complex 1	16 ± 0.76	14 ± 1.04	15 ± 0.87	12 ± 0.43	13 ± 0.38	12 ± 0.07	0.0 ± 0.0	9 ± 0.19
	A	A	A	A	A	A	A	A
Complex 2	11 ± 0.57	9 ± 0.28	11 ± 0.5	12 ± 0.58	12 ± 0.09	11 ± 0.31	11 ± 0.12	0.0 ± 0.0
	B	B	A	A	A	A	B	B
Complex 3	13 ± 1.0	12 ± 0.86	18 ± 1.15	14 ± 0.14	13 ± 0.20	16 ± 0.52	9 ± 0.05	9 ± 0.34
	B	A	B	A	A	B	C	A
Standard								
Ampicillin antibacterial agent	26 ± 0.49	21 ± 0.69	27 ± 0.53	25 ± 0.23	28 ± 0.79	26 ± 0.26	–	–
	C	C	C	B	B	C		
Amphotericin B	–	–	–	–	–	–	17 ± 0.50	21 ± 0.01
Antifungal agent							D	C

Different symbols indicate significant (*p* < 0.05) different means

G, gram reaction

Complexes did not produce differences in inhibition zones for Gram-negative *Escherichia coli*. A larger inhibition zone was induced by complex three (16 ± 0.52 mm) against Pseudomonas aeruginosa than by complexes one and two (12 ± 0.07 and 11 ± 0.31 mm, respectively). The latter inhibition zones were not significantly different. Non-significant differences in inhibition zone diameters among complexes were observed for Neisseria Gonorrhoeae.

Complex one demonstrated notable antibacterial activity against *B. subtilis* and *S. aureus,* consistent with Amoah et al. [138]. These authors suggested that metal complexes penetrate and disrupt bacterial cell walls [139]. Compared to Gram-negative bacteria, Gram-positive bacteria were more susceptible to the palladium complex. This activity may be attributed to the structure of bacterial cell walls [56]. The cell wall of Gram-positive bacteria has many layers of peptidoglycan. Bacteria cannot survive without a cell wall, and certain antibiotics kill bacteria by blocking peptidoglycan synthesis. Conversely, Gram-negative bacteria have thinner peptidoglycan cell walls and a second membrane formed by lipopolysaccharides and lipoproteins [140]. These differences in cell wall structure lead to differences in antibacterial susceptibility; certain antibiotics are exclusively effective against Gram-positive bacteria, and others are ‏ineffective [141].

The antibacterial activity of complexes varies depending on microbial cell permeability [142]. Regarding Pseudomonas aeruginosa, complexes one and two demonstrate similar antibacterial activity and no significant differences were noted in activity among complexes against *E. coli* and *N. Gonorrhoeae*. The antibacterial activity of complex three is superior against *P. aeruginosa*. Complex three is also most effective against *Str. faecalis* might be effective in treating diseases caused by this organism. Copper complexes two and three show activity against *P. aeruginosa* and *E. coli*, consistent with Adimule et al. [143]. Electron delocalization upon complexation throughout the chelate ring may underlie the antibacterial activity of metal complexes; lipophilicity and penetration across cell membranes are enhanced by chelation [144].

#### Antifungal activity

**Fig. 24:**
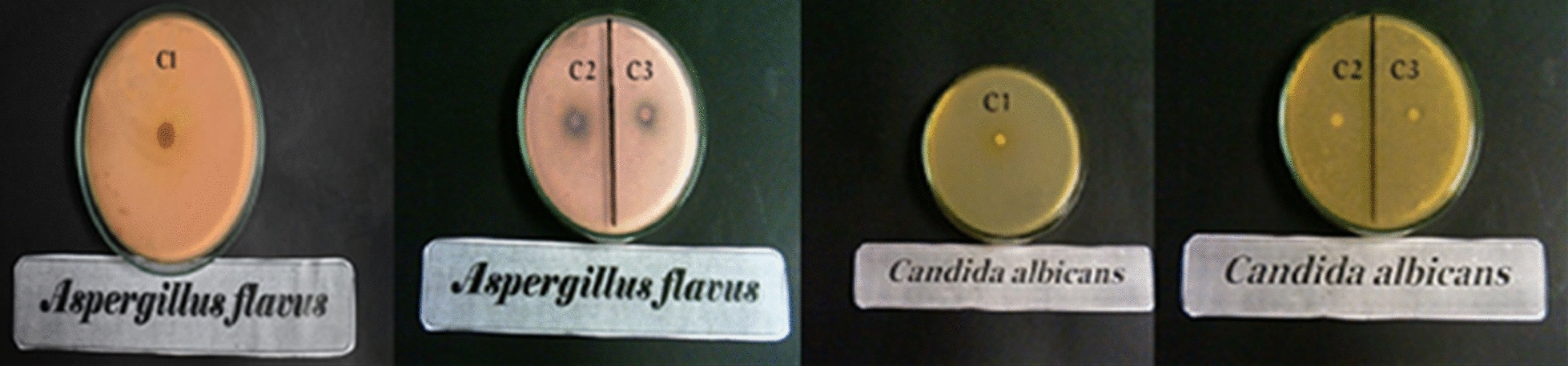
The antifungal activity of complexes

**Fig. 25:**
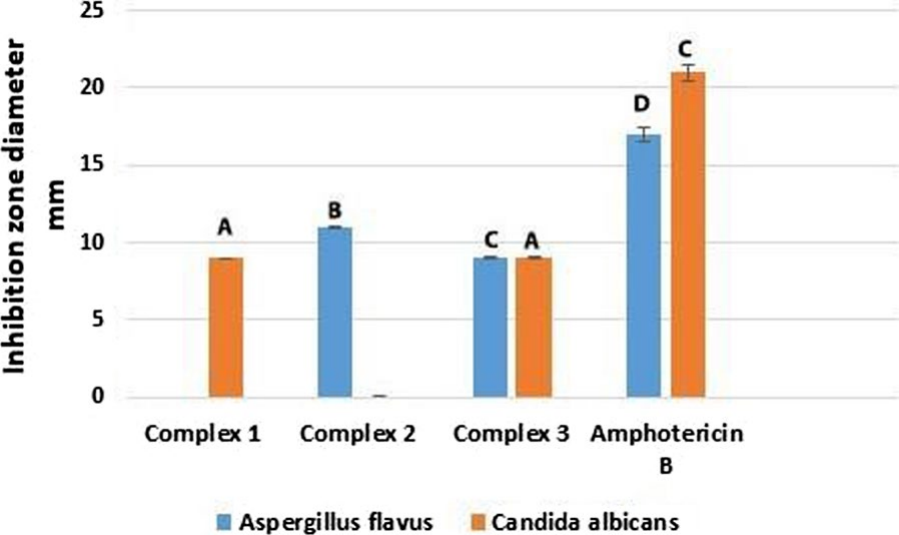
In vitro antifungal activity of complexes regarding inhibition zone diameter in mm. Different symbols indicate significant (*p* < 0.05) different means

Antifungal activity was assessed using two fungal strains with amphotericin B as a reference drug and DMSO as a solvent that does not inhibit fungal growth. Again, inhibition zones were measured millimeters (see Table 10 and Figs. 24, 25). Complex two induced a larger inhibition zone against *Aspergillus flavus* (11 ± 0.12 mm) than complexes one and three (0.0 ± 0.0 mm and 9 ± 0.05, respectively). Conversely, complexes one and three show larger inhibition zones (9 ± 0.19 and 9 ± 0.34 mm, respectively) than complex two, with zero inhibition zone against *Candida albicans*. According to these results, it can be deduced that complexes one and three give weak activity against *Candida albicans,* and complex two does not have any effect (activity) on *Candida albicans*.

Copper complexes show better activity against *Aspergillu*s *flavus*, consistent with Abdelkarim et al. [56], and palladium chelate does not display activity against *Aspergillus flavus*. Thus, activity depends on the metal ion [55] and possibly on the type of ligand. Adsorption of metal ions on microbial cell walls disrupts respiration and consequently prevents the synthesis of essential proteins, suggesting that growth inhibition necessitates the presence of metal ions [145]. Metal chelates have a higher level of lipophilicity that may explain improved activity against some fungi [146]. Some metal complexes show no activity, perhaps due to their insolubility in the medium or inability to penetrate the cell membrane [147]. Hence, antifungal action is influenced by lipophilicity, which facilitates penetration across lipid membranes [145]. Gałczyńska et al. [148] synthesized the following complexes: [Cu(iaa)_2_H_2_O](1), [Co(iaa)_2_(H_2_O)_2_]·H_2_O(2), [Cu(1-allim)_4_(NO_3_)_2_])(3) and [Co(1-allim)_6_] (NO3)_2_(4)where (iaa) = imidazole-4-acetate anion and (1-allim) = 1-allylimidazole. The antibacterial and antifungal activities of the complexes were tested against strains of S. aureus, E. coli, P. aeruginosa, and Candida albicans. Results showed that complex 3 [Cu(1-allim)4(NO3)2] and complex 2 [Co(iaa)_2_(H_2_O)_2_].H_2_O may be used as antifungal drugs.

## Conclusion

AMI complexes one, two, and three have been successfully prepared and characterized. Complexes characterization showed that the AMI ligand behaved as a neutral bidentate ligand coordinated with metal ions via imidazole and NH_2_ nitrogens. In contrast, malonic and oxalic acids functioned as uni-negative, bidentate ligands via carboxylate O and amino N. The anticancer activity study results reveal that complex two (Cu(AMI)L^1^) may be a potential pharmacological drug for breast cancer treatment. Complex two induced the most severe DNA damage in both cancer cell lines. Treatment of cells with complex one (palladium complex) decreased catalase activity. Complex one also demonstrated the greatest antioxidant activity. Gel electrophoresis results revealed complex two has a comparable impact on HeLa and MCF-7 treated cells. Antimicrobial assays identified complex three might be an effective antibacterial agent against *Streptococcus faecalis*.

## Supplementary Information

**Additional file 1. Table S1**: The optimized molecular orbital parameters of [Pd(AMI)Cl_2_], where AMI = amino methyl imidazole. **Table S2**: The optimized molecular orbital parameters of [Cu(AMI)L^1^], where AMI = amino methyl imidazole, L1 = oxalate.

## Data Availability

All data needed to support the conclusions are included in this article. Additional data related to this paper can be requested from the author (hfahmy@sci.cu.edu.eg).

## References

[1] Mjos KD, Orvig C (2014). Metallodrugs in medicinal inorganic chemistry. Chem Rev.

[2] Kamelan Kafi M, Bolvari NE, Mohammad Pour S, Moghadam SK, Shafaei N, Karimi E, Oskoueian E (2022). Encapsulated phenolic compounds from Ferula gummosa leaf: a potential phytobiotic against Campylobacter jejuni infection. J Food Process Preserv.

[3] World Health Organization. WHO report on cancer: setting priorities, investing wisely and providing care for all; 2020.

[4] Da Silva JP, Fuganti O, Kramer MG, Facchin G, Aquino LE, Ellena J, de Araujo MP (2020). Electrochemical, mechanistic, and DFT studies of amine derived diphosphines containing Ru(II)–cymene complexes with potent in vitro cytotoxic activity against HeLa and triple-negative breast cancer cells MDA-MB-231. Dalton Trans.

[5] Jaividhya P, Dhivya R, Akbarsha MA, Palaniandavar M (2012). Efficient DNA cleavage mediated by mononuclear mixed ligand copper(II) phenolate complexes: the role of co-ligand planarity on DNA binding and cleavage and anticancer activity. J Inorg Biochem.

[6] Veselov A, Burger RM, Scholes CP (1998). Q-band electron nuclear double resonance of ferric bleomycin and activated bleomycin complexes with DNA: Fe(III) hyperfine interaction with 31P and DNA-induced perturbation to bleomycin structure. J Am Chem Soc.

[7] Maheswari PU, Lappalainen K, Sfregola M, Barends S, Gamez P, Turpeinen U, Reedijk J (2007). Structure and DNA cleavage properties of two copper(II) complexes of the pyridine-pyrazole-containing ligands mbpzbpy and Hmpzbpya. Dalton Trans.

[8] Chitrapriya N, Shin JH, Hwang IH, Kim Y, Kim C, Kim SK (2015). Synthesis, DNA binding profile and DNA cleavage pathway of divalent metal complexes. RSC Adv.

[9] Wong E, Giandomenico CM (1999). Current status of platinum-based antitumor drugs. Chem Rev.

[10] Giaccone G (2000). Clinical perspectives on platinum resistance. Drugs.

[11] Johnson NP, Butour JL, Villani G, Wimmer FL, Defais M, Pierson V, Brabec V. Metal antitumor compounds: the mechanism of action of platinum complexes. In *Ruthenium and other non-platinum metal complexes in cancer chemotherapy*. Berlin: Springer; 1989. p. 1–24.

[12] Cohen SM, Lippard SJ. Cisplatin: from DNA damage to cancer chemotherapy; 2001. 10.1016/s0079-6603(01)67026-0 11525387

[13] Jamieson ER, Lippard SJ (1999). Structure, recognition, and processing of cisplatin—DNA adducts. Chem Rev.

[14] Kartalou M, Essigmann JM (2001). Mechanisms of resistance to cisplatin. Mut Res Fundam Mol Mech Mut.

[15] Brabec V. DNA modify*cations by antitumor platinum and ruthenium compounds: their recognition and repair; 2002. 10.1016/s0079-6603(02)71040-4 12102553

[16] Sundquist WI, Lippard SJ (1990). The coordination chemistry of platinum anticancer drugs and related compounds with DNA. Coord Chem Rev.

[17] Dalbiès R, Payet D, Leng M (1994). DNA double helix promotes a linkage isomerization reaction in trans-diamminedichloroplatinum(II)-modified DNA. Proc Natl Acad Sci.

[18] Budzisz E (2019). Role of metal ions complexes and their ligands in medicine pharmacy and cosmetology. Curr Med Chem.

[19] Kamal T, Khan SB, Haider S, Alghamdi YG, Asiri AM (2017). Thin layer chitosan-coated cellulose filter paper as substrate for immobilization of catalytic cobalt nanoparticles. Int J Biol Macromol.

[20] Fulton JR, Holland AW, Fox DJ, Bergman RG (2002). Formation, reactivity, and properties of nondative late transition metal–oxygen and—nitrogen bonds. Acc Chem Res.

[21] Hartwig JF (2008). Carbon–heteroatom bond formation catalyzed by organometallic complexes. Nature.

[22] Sohrabi M, Saeedi M, Larijani B, Mahdavi M (2021). Recent advances in biological activities of rhodium complexes: their applications in drug discovery research. Eur J Med Chem.

[23] Burchenal JH, Kalaher K, Dew K, Lokys L (1979). Rationale for development of platinum analogs. Cancer Treat Rep.

[24] Abdel-Rahman LH, Abu-Dief AM, Ismael M, Mohamed MA, Hashem NA (2016). Synthesis, structure elucidation, biological screening, molecular modeling and DNA binding of some Cu(II) chelates incorporating imines derived from amino acids. J Mol Struct.

[25] Abdel-Rahman LH, Abu-Dief AM, El-Khatib RM, Abdel-Fatah SM (2016). Sonochemical synthesis, DNA binding, antimicrobial Evaluation and in vitro anticancer activity of three new nano-sized Cu(II), Co(II) and Ni(II) chelates based on tri-dentate NOO imine ligands as precursors for metal oxides. J Photochem Photobiol, B.

[26] Mohamad ADM, Abualreish MJA, Abu-Dief AM (2019). Antimicrobial and anticancer activities of cobalt(III)-hydrazone complexes: Solubilities and chemical potentials of transfer in different organic co-solvent-water mixtures. J Mol Liq.

[27] Rohand T, Ben EL, Ayouchia H, Achtak H, Ghaleb A, Derin Y, Tutar A, Tanemura K (2021). Design, synthesis, DFT calculations, molecular docking and antimicrobial activities of novel cobalt, chromium metal complexes of heterocyclic moiety-based 1,3,4-oxadiazole derivatives. J Biomol Struct Dyn.

[28] Celen S, Gungor E, Kara H, Azaz AD (2013). Synthesis, spectroscopic characterization, and antimicrobial activities of Ni(II) and Fe(II) complexes with N-(2-hydroxyethyl)-5-nitrosalicylaldimine. J Coordinat Chem.

[29] Kelland L (2007). The resurgence of platinum-based cancer chemotherapy. Nat Rev Cancer.

[30] Trudu F, Amato F, Vaňhara P, Pivetta T, Peña-Méndez EM, Havel J (2015). Coordination compounds in cancer: past, present and perspectives. J Appl Biomed.

[31] Qi L, Luo Q, Zhang Y, Jia F, Zhao Y, Wang F (2019). Advances in toxicological research of the anticancer drug cisplatin. Chem Res Toxicol.

[32] Simpson PV, Desai NM, Casari I, Massi M, Falasca M (2019). Metal-based antitumor compounds: beyond cisplatin. Future Med Chem.

[33] Zhang JJ, Lu W, Sun RWY, Che CM (2012). Organogold(III) supramolecular polymers for anticancer treatment. Angew Chem Int Ed.

[34] Hu D, Liu Y, Lai YT, Tong KC, Fung YM, Lok CN, Che CM (2016). Anticancer gold(III) porphyrins target mitochondrial chaperone Hsp60. Angew Chem Int Ed.

[35] Fung SK, Zou T, Cao B, Lee PY, Fung YME, Hu D, Che CM (2017). Cyclometalated gold(III) complexes containing N-heterocyclic carbene ligands engage multiple anti-cancer molecular targets. Angew Chem.

[36] Zhang P, Sadler PJ (2017). Advances in the design of organometallic anticancer complexes. J Organomet Chem.

[37] Englinger B, Pirker C, Heffeter P, Terenzi A, Kowol CR, Keppler BK, Berger W (2018). Metal drugs and the anticancer immune response. Chem Rev.

[38] Wragg D, De Almeida A, Bonsignore R, Kühn FE, Leoni S, Casini A (2018). On the mechanism of Gold/NHC compounds binding to DNA G-quadruplexes: combined metadynamics and biophysical methods. Angew Chem.

[39] Zou T, Lok CN, Wan PK, Zhang ZF, Fung SK, Che CM (2018). Anticancer metal-N-heterocyclic carbene complexes of gold, platinum and palladium. Curr Opin Chem Biol.

[40] Ranford JD, Sadler PJ, Tocher DA (1993). Cytotoxicity and antiviral activity of transition-metal salicylato complexes and crystal structure of bis(diisopropylsalicylato)(1,10-phenanthroline) copper(II). J Chem Soc Dalton Trans.

[41] Zoroddu MA, Zanetti S, Pogni R, Basosi R (1996). An electron spin resonance study and antimicrobial activity of copper(II)-phenanthroline complexes. J Inorg Biochem.

[42] Garoufis A, Hadjikakou SK, Hadjiliadis NJCCR (2009). Palladium coordination compounds as anti-viral, anti-fungal, anti-microbial and anti-tumor agents. Coord Chem Rev.

[43] Ghani NTA, Mansour AM (2011). Novel Pd(II) and Pt(II) complexes of N,N-donor benzimidazole ligand: synthesis, spectral, electrochemical, DFT studies and evaluation of biological activity. Inorg Chim Acta.

[44] Heydari M, Moghadam ME, Tarlani A, Farhangian H (2017). DNA as a target for anticancer phen-imidazole Pd(II) complexes. Appl Biochem Biotechnol.

[45] Caudle MT, Kampf JW, Kirk ML, Rasmussen PG, Pecoraro VL (1997). The first binuclear Mn(IV) complex containing a bridging imidazolate ligand exhibits unique EPR spectral features. J Am Chem Soc.

[46] Colacio E, Ghazi M, Kivekäs R, Klinga M, Lloret F, Moreno JM (2000). A rational design for imidazolate-bridged linear trinuclear compounds from mononuclear Copper(II) complexes with 2-[((Imidazol-2-ylmethylidene) amino) ethyl] pyridine (HL): syntheses, structures, and magnetic properties of [Cu (L)(hfac) M (hfac) 2Cu (hfac)(L)](M= ZnII, CuII, MnII). Inorg Chem.

[47] Ishak NN, Jamsari J, Ismail AZ, Tahir MI, Tiekink ER, Veerakumarasivam A, Ravoof TB (2019). Synthesis, characterisation and biological studies of mixed-ligand nickel(II) complexes containing imidazole derivatives and thiosemicarbazide Schiff bases. J Mol Struct.

[48] Puratchikody A, Doble M (2007). Antinociceptive and antiinflammatory activities and QSAR studies on 2-substituted-4,5-diphenyl-1*H*-imidazoles. Bioorg Med Chem.

[49] Dutta S (2010). Synthesis and anthelmintic activity of some novel 2-substituted-4,5-diphenyl imidazoles. Acta Pharm.

[50] Khan MS, Hayat MU, Khanam M, Saeed H, Owais M, Khalid M, Ahmad M (2021). Role of biologically important imidazole moiety on the antimicrobial and anticancer activity of Fe(III) and Mn(II) complexes. J Biomol Struct Dyn.

[51] Abdel-Rahman LH, Basha MT, Al-Farhan BS, Ismael M (2022). Synthesis, biological assay, chemical descriptors, and molecular docking calculations of novel copper(II) mixed-ligand complexes of *n*-benzoyl-dl-phenylalanine and n-heterocyclic nitrogen bases. J Mol Struct.

[52] Fnfoon DY, Al-Adilee KJ (2023). Synthesis and spectral characterization of some metal complexes with new heterocyclic azo imidazole dye ligand and study biological activity as anticancer. J Mol Struct.

[53] El-Sherif AA, Shoukry MM, Abobakr LO (2013). Bivalent transition metal complexes of cetirizine: spectroscopic, equilibrium studies and biological activity. Spectrochim Acta Part A Mol Biomol Spectrosc.

[54] El-Sherif AA, Shoukry MM, Abd Elkarim AT, Barakat MH (2014). Protonation equilibria of biologically active ligands in nonaqueous media. Bioinorgan Chem Appl.

[55] El-Sherif AA, Shehata MR, Shoukry MM, Mahmoud NM (2018). Equilibrium studies of diethyltin(IV) dichloride and divinyltin(IV) dichloride with 1-(2-aminoethyl)-pyrolidine. J Mol Liq.

[56] Abdelkarim AT, Al-Shomrani MM, Rayan AM, El-Sherif AA (2015). Mixed ligand complex formation of cetirizine drug with bivalent transition metal(II) ions in the presence of 2-aminomethylbenzimidazole: synthesis, structural, biological, pH-metric, and thermodynamic studies. J Solution Chem.

[57] Abdelkarim AT, Mahmoud WH, El-Sherif AA (2021). Potentiometric, thermodynamics and coordination properties for binary and mixed ligand complexes of copper(II) with cephradine antibiotic and some N- and O-bound amino acids (α-alanine and β-alanine). J Mol Liq.

[58] Asla KA, Abdelkarimm AT, El-Reash GMA, El-Sherif AA (2020). Potentiometric, thermodynamics and DFT calculations of some metal(II)-schiff base complexes formed in solution. Int J Electrochem Sci.

[59] Hsieh HP, Liou JP, Lin YT, Mahindroo N, Chang JY, Yang YN, Wang CC (2003). Structure–activity and crystallographic analysis of benzophenone derivatives—the potential anticancer agents. Bioorg Med Chem Lett.

[60] Subbaraj P, Ramu A, Raman N, Dharmaraja J (2015). Synthesis, characterization, DNA interaction and pharmacological studies of substituted benzophenone derived Schiff base metal(II) complexes. J Saudi Chem Soc.

[61] Abu-Dief AM, Abdel-Rahman LH, Abdelhamid AA, Marzouk AA, Shehata MR, Bakheet MA, Nafady A (2020). Synthesis and characterization of new Cr(III), Fe(III) and Cu(II) complexes incorporating multi-substituted aryl imidazole ligand: Structural, DFT, DNA binding, and biological implications. Spectrochim Acta Part A Mol Biomol Spectrosc.

[62] Kumar G, Devi S, Kumar D (2016). Synthesis of Schiff base 24-membered trivalent transition metal derivatives with their anti-inflammation and antimicrobial Evaluation. J Mol Struct.

[63] Aljahdali MS, El-Sherif AA (2020). Synthesis and biological evaluation of novel Zn(II) and Cd(II) Schiff base complexes as antimicrobial, antifungal, and antioxidant agents. Bioinorgan Chem Appl.

[64] Ganeshpandian M, Loganathan R, Ramakrishnan S, Riyasdeen A, Akbarsha MA, Palaniandavar M (2013). Interaction of mixed ligand copper(II) complexes with CT DNA and BSA: effect of primary ligand hydrophobicity on DNA and protein binding and cleavage and anticancer activities. Polyhedron.

[65] El-Sherif AA, Shoukry MM, Abd Elkarim AT, Barakat MH (2014). Protonation equilibria of biologically active ligands in mixed aqueous organic solvents. Bioinorgan Chem Appl.

[66] Kareem MJ, Al-Hamdani AAS, Ko YG, Al Zoubi W, Mohammed SG (2021). Synthesis, characterization, and determination antioxidant activities for new Schiff base complexes derived from 2-(1*H*-indol-3-yl)-ethylamine and metal ion complexes. J Mol Struct.

[67] Cory AH, Owen TC, Barltrop JA, Cory JG (1991). Use of an aqueous soluble tetrazolium/formazan assay for cell growth assays in culture. Cancer Commun.

[68] Berridge MV, Herst PM, Tan AS (2005). Tetrazolium dyes as tools in cell biology: new insights into their cellular reduction. Biotechnol Annu Rev.

[69] Karimi E, Oskoueian E, Karimi A, Noura R, Ebrahimi M (2018). *Borago officinalis* L. flower: a comprehensive study on bioactive compounds and its health-promoting properties. J Food Meas Charact.

[70] Monks A, Scudiero D, Skehan P, Shoemaker R, Paull K, Vistica D, Boyd M (1991). Feasibility of a high-flux anticancer drug screen using a diverse panel of cultured human tumor cell lines. JNCI J Natl Cancer Inst.

[71] Rahmani F, Karimi E, Oskoueian E (2020). Synthesis and characterisation of chitosan-encapsulated genistein: its anti-proliferative and anti-angiogenic activities. J Microencapsul.

[72] Bauer AW, Kirby WM, Sherris C, Turck M (1966). Antibiotic susceptibility testing by a standardized single disk method. Am J Clin Pathol.

[73] National Committee for Clinical Laboratory Standards. Methods for dilution antimicrobial susceptibility tests for bacteria that grow aerobically. Approved standard M7-A3. National Committee for Clinical Laboratory Standards, Villanova, PA; 1993.

[74] Hall MD, Failes TW, Yamamoto N, Hambley TW (2007). Bioreductive activation and drug chaperoning in cobalt pharmaceuticals. Dalton Trans.

[75] Liebowitz LD, Ashbee HR, Evans EGV, Chong Y, Mallatova N, Zaidi M, Global Antifungal Surveillance Group (2001). A two year global evaluation of the susceptibility of Candida species to fluconazole by disk diffusion. Diagnostic Microbiol Infect Dis.

[76] Matar MJ, Ostrosky-Zeichner L, Paetznick VL, Rodriguez JR, Chen E, Rex JH (2003). Correlation between E-test, disk diffusion, and microdilution methods for antifungal susceptibility testing of fluconazole and voriconazole. Antimicrob Agents Chemother.

[77] Fossati P, Prencipe L, Berti G (1980). Use of 3,5-dichloro-2-hydroxybenzenesulfonic acid/4-aminophenazone chromogenic system in direct enzymic assay of uric acid in serum and urine. Clin Chem.

[78] Aebi H (1984). Catalase in vitro. Meth Enzymol.

[79] Fahmy HM, Ebrahim NM, Gaber MH (2020). In-vitro Evaluation of copper/copper oxide nanoparticles cytotoxicity and genotoxicity in normal and cancer lung cell lines. J Trace Elem Med Biol.

[80] Ruiz-Larrea MB, Leal AM, Liza M, Lacort M, de Groot H (1994). Antioxidant effects of estradiol and 2-hydroxyestradiol on iron-induced lipid peroxidation of rat liver microsomes. Steroids.

[81] Zarei M, Karimi E, Oskoueian E, Es-Haghi A, Yazdi MET (2021). Comparative study on the biological effects of sodium citrate-based and apigenin-based synthesized silver nanoparticles. Nutr Cancer.

[82] Kesavan MP, Kumar GV, Raja JD, Anitha K, Karthikeyan S, Rajesh J (2017). DNA interaction, antimicrobial, antioxidant and anticancer studies on Cu(II) complexes of Luotonin A. J Photochem Photobiol B.

[83] Chen Z, Bertin R, Froldi G (2013). EC50 estimation of antioxidant activity in DPPH assay using several statistical programs. Food Chem.

[84] Singh NP, McCoy MT, Tice RR, Schneider EL (1988). A simple technique for quantitation of low levels of DNA damage in individual cells. Exp Cell Res.

[85] Shao J, Ma ZY, Li A, Liu YH, Xie CZ, Qiang ZY, Xu JY (2014). Thiosemicarbazone Cu(II) and Zn(II) complexes as potential anticancer agents: Syntheses, crystal structure, DNA cleavage, cytotoxicity and apoptosis induction activity. J Inorg Biochem.

[86] Stepanenko AA, Dmitrenko VV (2015). Pitfalls of the MTT assay: direct and off-target effects of inhibitors can result in over/underestimation of cell viability. Gene.

[87] Lopes UG, Erhardt P, Yao R, Cooper GM (1997). p53-dependent induction of apoptosis by proteasome inhibitors. J Biol Chem.

[88] Lee RF, Steinert S (2003). Use of the single cell gel electrophoresis/comet assay for detecting DNA damage in aquatic (marine and freshwater) animals. MutRes/Rev Mut Res.

[89] Fairbairn DW, Olive PL, O’Neill KL (1995). The comet assay: a comprehensive review. Mut Res/Rev Genet Toxicol.

[90] Klaude M, Eriksson S, Nygren J, Ahnström G (1996). The comet assay: mechanisms and technical considerations. Mut Res/DNA Repair.

[91] Anderson D, Yu TW, McGregor DB (1998). Comet assay responses as indicators of carcinogen exposure. Mutagenesis.

[92] Arjmand F, Muddassir M, Zaidi Y, Ray D (2013). Design, synthesis and crystal structure determination of dinuclear copper-based potential chemotherapeutic drug entities; in vitro DNA binding, cleavage studies and an evaluation of genotoxicity by micronucleus test and comet assay. Med Chem Commun.

[93] Kassie F, Parzefall W, Knasmüller S (2000). Single cell gel electrophoresis assay: a new technique for human biomonitoring studies. Mut Res/Rev Mut Res.

[94] Tice RR, Agurell E, Anderson D, Burlinson B, Hartmann A, Kobayashi H, Sasaki YF (2000). Single cell gel/comet assay: guidelines for in vitro and in vivo genetic toxicology testing. Environ Mol Mutagen.

[95] Tchounwou CK, Yedjou CG, Farah I, Tchounwou PB (2014). d-Glucose-induced cytotoxic, genotoxic, and apoptotic effects on human breast adenocarcinoma (MCF-7) cells. J Cancer Sci Ther.

[96] Bortner CD, Oldenburg NBE, Cidlowski JA (1995). The role of DNA fragmentation in apoptosis. Trends Cell Biol.

[97] El-Sherif AA, Fetoh A, Abdulhamed YK, El-Reash GMA (2018). Synthesis, structural characterization, DFT studies and biological activity of Cu(II) and Ni(II) complexes of novel hydrazone. Inorg Chim Acta.

[98] Elhagali GAM, Elsayed GA, Eliswey RA, El-Sherif AA (2018). Molecular modeling and cyclization reactions of 2-(4-oxothiazolidine-2-ylidene) acetonitrile. J Iran Chem Soc.

[99] Adly OM, El-Shafiy HF, Shebl M (2019). Synthesis, spectroscopic studies, DFT calculations, antimicrobial and antitumor activity of tridentate NNO Schiff base metal complexes based on 5-acetyl-4-hydroxy-2H-1, 3-thiazine-2, 6 (3H)-dione. J Mol Struct.

[100] Shebl M (2017). Coordination behavior of new bis (tridentate ONO, ONS and ONN) donor hydrazones towards some transition metal ions: synthesis, spectral, thermal, antimicrobial and antitumor studies. J Mol Struct.

[101] Portakal ED, Yeliz K, Emire D, Elif KY, Aye E, Ismet (2021). Kaya_3Appl organomet. Chem.

[102] Zhou QQ, Miao RQ, Wang DF, Huang RB (2020). Syntheses, structures and properties of three novel Cu(II) coordination compounds based on 4,4′-oxybisbenzoic acid. J Mol Struct.

[103] Dhanaraj CJ, Raj SS (2020). Synthesis, characterization and biological studies of Schiff base metal complexes derived from 4-aminoantipyrine, acetamide and p-phenylenediamine. Inorg Chem Commun.

[104] Srinivasan S, Athappan P, Rajagopal G (2001). Synthesis, spectral and redox properties of metal complexes of macrocyclic tetraaza chiral Schiff bases. Trans Met Chem.

[105] Suvarapu LN, Somala AR, Koduru JR, Baek SO, Ammireddy VR (2012). A critical review on analytical and biological applications of thio-and phenylthiosemicarbazones. Asian J Chem.

[106] Shit S, Sasmal A, Dhal P, Rizzoli C, Mitra S (2016). J Mol Struct.

[107] Abdel-Rahman LH, Adam MS, Abu-Dief AM, Ahmed HES, Nafady A (2020). Non-linear optical property and biological assays of therapeutic potentials under in vitro conditions of Pd(II), Ag(I) and Cu(II) complexes of 5-diethyl amino-2-({2-[(2-hydroxy-benzylidene)-amino]-phenylimino}-methyl)-phenol. Molecules.

[108] Tyagi N, Viji M, Karunakaran SC, Varughese S, Ganesan S, Priya S, Ramaiah D (2015). Enhancement in intramolecular interactions and in vitro biological activity of a tripodal tetradentate system upon complexation. Dalton Trans.

[109] Ma T, Xu J, Wang Y, Yu H, Yang Y, Liu Y, Zhu T (2015). Ternary copper(II) complexes with amino acid chains and heterocyclic bases: DNA binding, cytotoxic and cell apoptosis induction properties. J Inorg Biochem.

[110] Tweedy BG (1964). Plant extracts with metal ions as potential antimicrobial agents. Phytopathology.

[111] Rahman LHA, Abu-Dief AM, Hamdan SK, Seleem AA (2015). Nano structure Iron(II) and Copper(II) Schiff base complexes of a NNO-tridentate ligand as new antibiotic agents: spectral, thermal behaviors and DNA binding ability. Int J Nano Chem.

[112] Karami K, Hosseini-Kharat M, Sadeghi-Aliabadi H, Lipkowski J, Mirian M (2014). In vitro cytotoxicity studies of palladacyclic complexes containing the symmetric diphosphine bridging ligand. Studies of their interactions with DNA and BSA. Eur J Med Chem.

[113] Gündüz MK, Bolat M, Kaymak G, Berikten D, Köse DA (2021). Therapeutic effects of newly synthesized boron compounds (BGM and BGD) on hepatocellular carcinoma. Biol Trace Element Res.

[114] Roleira FM, Tavares-da-Silva EJ, Varela CL, Costa SC, Silva T, Garrido J, Borges F (2015). Plant derived and dietary phenolic antioxidants: anticancer properties. Food Chem.

[115] Petrasheuskaya TV, Kiss MA, Dömötör O, Holczbauer T, May NV, Spengler G, Enyedy ÉA (2020). Salicylaldehyde thiosemicarbazone copper complexes: impact of hybridization with estrone on cytotoxicity, solution stability and redox activity. New J Chem.

[116] Gupta RK, Patel AK, Shah N, Choudhary AK, Jha UK, Yadav UC, Pakuwal U (2014). Oxidative stress and antioxidants in disease and cancer: a review. Asian Pac J Cancer Prev.

[117] Li S, Tan HY, Wang N, Zhang ZJ, Lao L, Wong CW, Feng Y (2015). The role of oxidative stress and antioxidants in liver diseases. Int J Mol Sci.

[118] Altay A, Caglar S, Caglar B (2019). Silver(I) complexes containing diclofenac and niflumic acid induce apoptosis in human-derived cancer cell lines. Arch Physiol Biochem.

[119] Awad MG, Ali RA, Abd El-Monem DD, El-Magd MA (2020). Graviola leaves extract enhances the anticancer effect of cisplatin on various cancer cell lines. Mol Cell Toxicol.

[120] Gardner HW (1989). Oxygen radical chemistry of polyunsaturated fatty acids. Free Radical Biol Med.

[121] Niedernhofer LJ, Daniels JS, Rouzer CA, Greene RE, Marnett LJ (2003). Malondialdehyde, a product of lipid peroxidation, is mutagenic in human cells. J Biol Chem.

[122] Spiteller P, Kern W, Reiner J, Spiteller G (2001). Aldehydic lipid peroxidation products derived from linoleic acid. Biochim Biophys Acta (BBA) Mol Cell Biol Lipids.

[123] Janicka M, Kot-Wasik A, Kot J, Namieśnik J (2010). Isoprostanes-biomarkers of lipid peroxidation: their utility in evaluating oxidative stress and analysis. Int J Mol Sci.

[124] Ho E, Galougahi KK, Liu CC, Bhindi R, Figtree GA (2013). Biological markers of oxidative stress: applications to cardiovascular research and practice. Redox Biol.

[125] Khan RA, Khan MR, Usman M, Sayeed F, Alghamdi HA, Alrumman S, Alsalme A (2020). β-Carboline copper complex as a potential mitochondrial-targeted anticancer chemotherapeutic agent: favorable attenuation of human breast cancer MCF7 cells via apoptosis. Saudi J Biol Sci.

[126] Alharbi W, Hassan I, Khan RA, Parveen S, Alharbi KH, Bin-Sharfan II, Alsalme A (2021). Bioactive tryptophan-based copper complex with auxiliary β-carboline spectacle potential on human breast cancer cells: in vitro and in vivo studies. Molecules.

[127] Lesiów MK, Pietrzyk P, Kyzioł A, Komarnicka UK (2019). Cu(II) complexes with FomA protein fragments of *Fusobacterium nucleatum* increase oxidative stress and malondialdehyde level. Chem Res Toxicol.

[128] Begin ME, Ells G, Horrobin DF (1988). Polyunsaturated fatty acid-induced cytotoxicity against tumor cells and its relationship to lipid peroxidation. JNCI J Natl Cancer Inst.

[129] Alzahrani FA, El-Magd MA, Abdelfattah-Hassan A, Saleh AA, Saadeldin IM, El-Shetry ES, Alkarim S (2018). Potential effect of exosomes derived from cancer stem cells and MSCs on progression of DEN-induced HCC in rats. Stem Cells Int.

[130] Elkeiy MM, Khamis AA, El-Gamal MM, Gazia MMA, Zalat ZA, El-Magd MA (2020). Chitosan nanoparticles from Artemia salina inhibit progression of hepatocellular carcinoma in vitro and in vivo. Environ Sci Pollut Res.

[131] Magdy A, Sadaka E, Hanafy N, El-Magd MA, Allahloubi N, El Kemary M (2020). Green tea ameliorates the side effects of the silver nanoparticles treatment of ehrlich ascites tumor in mice. Mol Cell Toxicol.

[132] Jirjees VY, Suleman VT, Ahmed SD, Al-Hamdani AAS (2020). Determination of antioxidant activity for metal ions complexes. J Duhok Univ.

[133] Manimaran P, Balasubramaniyan S, Azam M, Rajadurai D, Al-Resayes SI, Mathubala G, Khan I (2021). Synthesis, spectral characterization and biological activities of Co(II) and Ni(II) mixed ligand complexes. Molecules.

[134] Sadaf H, Zahra SS, Nadeem S, Tahir MN, Ahmad S, Andleeb S (2019). Synthesis, X-ray structures and biological properties of palladium(II) complexes of 1,2-dimethylimidazole and benzimidazole. Polyhedron.

[135] Pandey SK, Singh DP, Marverti G, Butcher RJ, Pratap S (2018). Monodentate coordination of N,N′-disubstituted thiocarbamide ligands: syntheses, structural analyses, in vitro cytotoxicity and DNA damage studies of Cu(I) complexes. ChemistrySelect.

[136] Aljohani ET, Shehata MR, Alkhatib F, Alzahrani SO, Abu-Dief AM (2021). Development and structure elucidation of new VO^2+^, Mn^2+^, Zn^2+^, and Pd-complexes based on azomethine ferrocenyl ligand: DNA interaction, antimicrobial, antioxidant, anticancer activities, and molecular docking. Appl Organomet Chem.

[137] Abu-Dief AM, El-Metwaly NM, Alzahrani SO, Alkhatib F, Abualnaja MM, El-Dabea T, Ali MAEAA (2021). Synthesis and characterization of Fe(III), Pd(II) and Cu(II)-thiazole complexes; DFT, pharmacophore modeling, in-vitro assay and DNA binding studies. J Mol Liq.

[138] Amoah C, Obuah C, Ainooson MK, Adokoh CK, Muller A (2021). Synthesis, characterization and antibacterial applications of pyrazolyl-sulfonamides and their palladium complexes. New J Chem.

[139] Santos AF, Brotto DF, Favarin LR, Cabeza NA, Andrade GR, Batistote M, Anjos AD (2014). Study of the antimicrobial activity of metal complexes and their ligands through bioassays applied to plant extracts. Rev Bras.

[140] El-Sherif AA, Eldebss TM (2011). Synthesis, spectral characterization, solution equilibria, in vitro antibacterial and cytotoxic activities of Cu(II), Ni(II), Mn(II), Co(II) and Zn(II) complexes with Schiff base derived from 5-bromosalicylaldehyde and 2-aminomethylthiophene. Spectrochim Acta Part A Mol Biomol Spectrosc.

[141] Koch AL (2003). The murein target for biological and medical attack: then, recently, now, and in the future. Clin Microbiol Rev.

[142] Abdel-Kader NS, Abdel-Latif SA, El-Ansary AL, Sayed AG (2021). Spectroscopic studies, density functional theory calculations, non-linear optical properties, biological activity of 1-hydroxy-4-((4-(N-(pyrimidin-2-yl) sulfamoyl) phenyl) diazenyl)-2-naphthoic acid and its chelates with Nickel(II), Copper(II), Zinc(II) and Palladium(II) metal ions. J Mol Struct.

[143] Adimule V, Yallur BC, Kamat V, Krishna PM (2021). Characterization studies of novel series of cobalt(II), nickel(II) and copper(II) complexes: DNA binding and antibacterial activity. J Pharm Investig.

[144] Sarker D, Hossen MF, Kudrat-E-Zahan M, Haque MM, Zamir R, Asraf MA (2020). Synthesis, characterization, thermal analysis and antibacterial activity of Cu(II) and Ni(II) complexes with thiosemicarbazone derived from thiophene-2-aldehyde. J Mater Sci Res Rev.

[145] Karim AT, El-Sherif AA (2014). Physicochemical studies and biological activity of mixed ligand complexes involving bivalent transition metals with a novel Schiff base and glycine as a representative amino acid. Eur J Chem.

[146] Samota MK, Seth G (2010). Synthesis, characterization, and antimicrobial activity of palladium(II) and platinum(II) complexes with 2-substituted benzoxazole ligands. Heteroatom Chem Int J Main Group Elements.

[147] Benns BG, Gingras BA, Bayley CH (1960). Antifungal activity of some thiosemicarbazones and their copper complexes. Appl Microbiol.

[148] Gałczyńska K, Ciepluch K, Madej Ł, Kurdziel K, Maciejewska B, Drulis-Kawa Z, Arabski M (2019). Selective cytotoxicity and antifungal properties of copper(II) and cobalt(II) complexes with imidazole-4-acetate anion or 1-allylimidazole. Sci Rep.

