# Supplementary material for: Study of novel bidentate heterocyclic amine-based metal complexes and their biological activities: cytotoxicity and antimicrobial activity evaluation
Source: BMC Chem. 2023 Jul 15;17(1):78. doi: 10.1186/s13065-023-00996-1 (PMC10349454; doi:10.1186/s13065-023-00996-1)
Supplement: Supplementary file 1 — Additional file 1. Table S1: The optimized molecular orbital parameters of [Pd(AMI)Cl2], where AMI = amino methyl imidazole. Table S2: The optimized molecular orbital parameters of [Cu(AMI)L1], where AMI = amino methyl imidazole, L1 = oxalate. [file 13065_2023_996_MOESM1_ESM.docx]

**Table.S1. The optimized molecular orbital parameters of [Pd(AMI)Cl_2_], where AMI= amino methyl imidazole.**

| N(8)-H(17) | 1.024 | 1.035 |
| --- | --- | --- |
| N(8)-H(16) | 1.021 | 1.035 |
| Pd(7)-Cl(10) | 2.366 |  |
| Pd(7)-Cl(9) | 2.403 |  |
| N(8)-Pd(7) | 2.392 |  |
| C(6)-H(15) | 1.1 | 1.113 |
| C(6)-H(14) | 1.107 | 1.113 |
| N(8)-C(6) | 1.479 | 1.468 |
| Pd(7)-N(5) | 2.259 |  |
| C(4)-H(13) | 1.08 | 1.1 |
| N(5)-C(4) | 1.388 | 1.358 |
| C(3)-H(12) | 1.083 | 1.1 |
| C(3)-C(4) | 1.378 | 1.42 |
| N(2)-H(11) | 1.016 | 1.05 |
| N(2)-C(3) | 1.386 | 1.364 |
| C(1)-C(6) | 1.508 | 1.497 |
| N(5)-C(1) | 1.335 | 1.358 |
| C(1)-N(2) | 1.361 | 1.364 |
| H(17)-N(8)-H(16) | 108.155 |  |
| H(17)-N(8)-Pd(7) | 100.916 |  |
| H(17)-N(8)-C(6) | 111.671 | 109.47 |
| H(16)-N(8)-Pd(7) | 115.718 |  |
| H(16)-N(8)-C(6) | 111.284 | 109.47 |
| Pd(7)-N(8)-C(6) | 108.692 |  |
| Cl(10)-Pd(7)-Cl(9) | 165.792 |  |
| Cl(10)-Pd(7)-N(8) | 103.889 |  |
| Cl(10)-Pd(7)-N(5) | 89.189 |  |
| Cl(9)-Pd(7)-N(8) | 89.809 |  |
| Cl(9)-Pd(7)-N(5) | 98.134 |  |
| N(8)-Pd(7)-N(5) | 75.007 |  |
| H(15)-C(6)-H(14) | 107.193 | 109.4 |
| H(15)-C(6)-N(8) | 107.761 |  |
| H(15)-C(6)-C(1) | 107.957 | 109.41 |
| H(14)-C(6)-N(8) | 112.328 |  |
| H(14)-C(6)-C(1) | 110.715 | 109.41 |
| N(8)-C(6)-C(1) | 110.682 |  |
| Pd(7)-N(5)-C(4) | 136.427 |  |
| Pd(7)-N(5)-C(1) | 112.156 |  |
| C(4)-N(5)-C(1) | 107.389 |  |
| H(13)-C(4)-N(5) | 121.142 | 116.5 |
| H(13)-C(4)-C(3) | 130.052 | 120 |
| N(5)-C(4)-C(3) | 108.769 | 123.5 |
| H(12)-C(3)-C(4) | 132.437 | 120 |
| H(12)-C(3)-N(2) | 121.774 | 113.5 |
| C(4)-C(3)-N(2) | 105.776 | 119 |
| H(11)-N(2)-C(3) | 125.393 | 118 |
| H(11)-N(2)-C(1) | 125.998 | 118 |
| C(3)-N(2)-C(1) | 108.606 | 124 |
| C(6)-C(1)-N(5) | 125.34 | 115.1 |
| C(6)-C(1)-N(2) | 124.575 | 125.3 |
| N(5)-C(1)-N(2) | 109.453 | 126 |
| C(6)-N(8)-Pd(7)-N(5) | 24.99 |  |
| C(6)-N(8)-Pd(7)-Cl(9) | 123.464 |  |
| C(6)-N(8)-Pd(7)-Cl(10) | -60.346 |  |
| H(16)-N(8)-Pd(7)-N(5) | 151.025 |  |
| H(16)-N(8)-Pd(7)-Cl(9) | -110.501 |  |
| H(16)-N(8)-Pd(7)-Cl(10) | 65.69 |  |
| H(17)-N(8)-Pd(7)-N(5) | -92.544 |  |
| H(17)-N(8)-Pd(7)-Cl(9) | 5.93 |  |
| H(17)-N(8)-Pd(7)-Cl(10) | -177.88 |  |
| Pd(7)-N(8)-C(6)-C(1) | -27.38 |  |

**Table.S2. The optimized molecular orbital parameters of [Cu(AMI)L^1^], where AMI= amino methyl imidazole, L^1^= oxalate.**

| C(12)-O(14) | 1.241 | 1.208 |
| --- | --- | --- |
| C(11)-O(13) | 1.24 | 1.208 |
| C(11)-C(12) | 1.573 | 1.5 |
| O(10)-C(11) | 1.356 | 1.338 |
| O(9)-C(12) | 1.353 | 1.338 |
| N(8)-H(21) | 1.021 | 1.035 |
| N(8)-H(20) | 1.021 | 1.035 |
| Cu(7)-O(10) | 1.897 |  |
| Cu(7)-O(9) | 1.918 |  |
| Cu(7)-N(8) | 2.137 |  |
| C(6)-H(19) | 1.098 | 1.113 |
| C(6)-H(18) | 1.098 | 1.113 |
| C(6)-N(8) | 1.492 | 1.468 |
| N(5)-Cu(7) | 1.983 | 1.303 |
| C(4)-H(17) | 1.078 | 1.1 |
| C(4)-N(5) | 1.395 | 1.358 |
| C(3)-H(16) | 1.078 | 1.1 |
| C(3)-C(4) | 1.383 | 1.42 |
| N(2)-H(15) | 1.012 | 1.05 |
| N(2)-C(3) | 1.406 | 1.364 |
| C(1)-C(6) | 1.513 | 1.497 |
| C(1)-N(5) | 1.338 | 1.358 |
| C(1)-N(2) | 1.373 | 1.364 |
| O(14)-C(12)-C(11) | 122.598 | 123 |
| O(14)-C(12)-O(9) | 124.524 | 122 |
| C(11)-C(12)-O(9) | 112.877 | 124.3 |
| O(13)-C(11)-C(12) | 123.037 | 123 |
| O(13)-C(11)-O(10) | 124.415 | 122 |
| C(12)-C(11)-O(10) | 112.549 | 124.3 |
| C(11)-O(10)-Cu(7) | 114.311 |  |
| C(12)-O(9)-Cu(7) | 113.554 |  |
| H(21)-N(8)-H(20) | 109.083 |  |
| H(21)-N(8)-Cu(7) | 102.701 |  |
| H(21)-N(8)-C(6) | 114.629 | 109.47 |
| H(20)-N(8)-Cu(7) | 102.55 |  |
| H(20)-N(8)-C(6) | 114.27 | 109.47 |
| Cu(7)-N(8)-C(6) | 112.347 |  |
| O(10)-Cu(7)-O(9) | 86.709 |  |
| O(10)-Cu(7)-N(8) | 90.243 |  |
| O(10)-Cu(7)-N(5) | 172.055 |  |
| O(9)-Cu(7)-N(8) | 176.872 |  |
| O(9)-Cu(7)-N(5) | 101.224 |  |
| N(8)-Cu(7)-N(5) | 81.83 |  |
| H(19)-C(6)-H(18) | 107.002 | 109.4 |
| H(19)-C(6)-N(8) | 109.852 |  |
| H(19)-C(6)-C(1) | 110.25 | 109.41 |
| H(18)-C(6)-N(8) | 111.01 |  |
| H(18)-C(6)-C(1) | 110.674 | 109.41 |
| N(8)-C(6)-C(1) | 108.063 |  |
| Cu(7)-N(5)-C(4) | 136.23 |  |
| Cu(7)-N(5)-C(1) | 115.501 |  |
| C(4)-N(5)-C(1) | 108.268 |  |
| H(17)-C(4)-N(5) | 121.228 | 116.5 |
| H(17)-C(4)-C(3) | 130.476 | 120 |
| N(5)-C(4)-C(3) | 108.296 | 123.5 |
| H(16)-C(3)-C(4) | 131.442 | 120 |
| H(16)-C(3)-N(2) | 122.466 | 113.5 |
| C(4)-C(3)-N(2) | 106.092 | 119 |
| H(15)-N(2)-C(3) | 125.471 | 118 |
| H(15)-N(2)-C(1) | 126.441 | 118 |
| C(3)-N(2)-C(1) | 108.088 | 124 |
| C(6)-C(1)-N(5) | 121.948 | 115.1 |
| C(6)-C(1)-N(2) | 128.793 | 125.3 |
| N(5)-C(1)-N(2) | 109.255 | 126 |
| O(10)-C(11)-C(12)-O(9) | -0.11 |  |
| O(10)-C(11)-C(12)-O(14) | 179.885 |  |
| O(13)-C(11)-C(12)-O(9) | 179.893 |  |
| O(13)-C(11)-C(12)-O(14) | -0.112 |  |
| Cu(7)-O(10)-C(11)-C(12) | 0.061 |  |
| Cu(7)-O(10)-C(11)-O(13) | -179.943 |  |
| Cu(7)-O(9)-C(12)-C(11) | 0.105 |  |
| Cu(7)-O(9)-C(12)-O(14) | -179.889 |  |
| N(5)-Cu(7)-O(10)-C(11) | 176.899 |  |
| N(8)-Cu(7)-O(10)-C(11) | -179.303 |  |
| O(9)-Cu(7)-O(10)-C(11) | -0.005 |  |
| N(5)-Cu(7)-O(9)-C(12) | -179.626 |  |
| N(8)-Cu(7)-O(9)-C(12) | 12.918 |  |
| O(10)-Cu(7)-O(9)-C(12) | -0.062 |  |
| N(5)-Cu(7)-N(8)-C(6) | 4.697 |  |
| N(5)-Cu(7)-N(8)-H(20) | -118.45 |  |
| N(5)-Cu(7)-N(8)-H(21) | 128.376 |  |
| O(9)-Cu(7)-N(8)-C(6) | 172.269 |  |
| O(9)-Cu(7)-N(8)-H(20) | 49.122 |  |
| O(9)-Cu(7)-N(8)-H(21) | -64.052 |  |
| O(10)-Cu(7)-N(8)-C(6) | -174.773 |  |
| O(10)-Cu(7)-N(8)-H(20) | 62.08 |  |
| O(10)-Cu(7)-N(8)-H(21) | -51.094 |  |
| C(1)-C(6)-N(8)-Cu(7) | -5.869 |  |
| C(1)-C(6)-N(8)-H(20) | 110.43 |  |
| C(1)-C(6)-N(8)-H(21) | -122.611 |  |
| H(18)-C(6)-N(8)-Cu(7) | -127.407 |  |
| H(18)-C(6)-N(8)-H(20) | -11.108 |  |
| H(18)-C(6)-N(8)-H(21) | 115.851 |  |
| H(19)-C(6)-N(8)-Cu(7) | 114.449 |  |
| H(19)-C(6)-N(8)-H(20) | -129.251 |  |
| H(19)-C(6)-N(8)-H(21) | -2.293 |  |
| C(1)-N(5)-Cu(7)-N(8) | -2.204 |  |
| C(1)-N(5)-Cu(7)-O(9) | 178.482 |  |
| C(1)-N(5)-Cu(7)-O(10) | 1.634 |  |
| C(4)-N(5)-Cu(7)-N(8) | 177.752 |  |
| C(4)-N(5)-Cu(7)-O(9) | -1.562 |  |
| C(4)-N(5)-Cu(7)-O(10) | -178.411 |  |
| C(3)-C(4)-N(5)-C(1) | 0.027 |  |
| C(3)-C(4)-N(5)-Cu(7) | -179.93 |  |
| H(17)-C(4)-N(5)-C(1) | -179.976 |  |
| H(17)-C(4)-N(5)-Cu(7) | 0.067 |  |
| N(2)-C(3)-C(4)-N(5) | 0.007 |  |
| N(2)-C(3)-C(4)-H(17) | -179.99 |  |
| H(16)-C(3)-C(4)-N(5) | 179.926 |  |
| H(16)-C(3)-C(4)-H(17) | -0.07 |  |
| C(1)-N(2)-C(3)-C(4) | -0.037 |  |
| C(1)-N(2)-C(3)-H(16) | -179.966 |  |
| H(15)-N(2)-C(3)-C(4) | -179.997 |  |
| H(15)-N(2)-C(3)-H(16) | 0.074 |  |
| N(2)-C(1)-C(6)-N(8) | -176.218 |  |
| N(2)-C(1)-C(6)-H(18) | -54.473 |  |
| N(2)-C(1)-C(6)-H(19) | 63.713 |  |
| N(5)-C(1)-C(6)-N(8) | 4.626 |  |
| N(5)-C(1)-C(6)-H(18) | 126.371 |  |
| N(5)-C(1)-C(6)-H(19) | -115.444 |  |
| N(2)-C(1)-N(5)-C(4) | -0.052 |  |
| N(2)-C(1)-N(5)-Cu(7) | 179.916 |  |
| C(6)-C(1)-N(5)-C(4) | 179.252 |  |
| C(6)-C(1)-N(5)-Cu(7) | -0.78 |  |
| N(5)-C(1)-N(2)-C(3) | 0.056 |  |
| N(5)-C(1)-N(2)-H(15) | -179.985 |  |
| C(6)-C(1)-N(2)-C(3) | -179.186 |  |
| C(6)-C(1)-N(2)-H(15) | 0.773 |  |
